# First-Principles Investigation of Lithium Titanate Oxide as an Anode Material in Li‑, Na‑, Mg‑, Ca-, and K‑Ion Batteries

**DOI:** 10.1021/acsomega.5c04533

**Published:** 2025-07-24

**Authors:** Abdul Majid, Ramla Ashfaq, Sawaira Tasawar, Mohammad Alkhedher, Sajjad Haider, Kamran Alam, Hira Azhar Cheema

**Affiliations:** † Department of Physics, 128417 University of Gujrat, Gujrat 50700, Pakistan; ‡ Mechanical and Industrial Engineering Department, 105947 Abu Dhabi University, Abu Dhabi 59911, United Arab Emirates; § Chemical Engineering Department, College of Engineering, King Saud University, P.O. Box 800, Riyadh 11421, Saudi Arabia; ∥ Department of Chemical Engineering, Materials Environment Sapienza, 9311 University of Rome Roma, 00185, Italy

## Abstract

The development of electrode material is a top priority to meet the requirements of high storage capacity, longer cyclic stability, and rapid transportation of ions in rechargeable metal-ion batteries. In this research, first-principles investigations are carried out to examine the suitability of lithium titanate oxide as an anode material in a series of metal-ion batteries, including Li-ion batteries (LIBs) and various multivalent-ion batteries such as Al-ion batteries (AIBs), Mg-ion batteries (MIBs), Ca-ion batteries (CIBs), and potassium-ion batteries (KIBs). The proposed material is comprehensively investigated to study the structural properties, thermal stability, metal atom storage capacity, and adsorption energy. The ab initio molecular dynamics (AIMD) simulation is used to ensure the thermal stability of the host material. The ideal anodic properties of the material are examined by modeling the adsorption of Li, Mg, Ca, K, and Al on the host material, thereby monitoring the exothermic reaction to explore its suitability for the relevant batteries. The calculated values of the storage capacity for LIBs, AIBs, MIBs, CIBs, and KIBs are 240 mAhg^–1^, 1131 mAhg^–1^, 1302 mAhg^–1^, 411 mAhg^–1^, 171 mAhg^–1^, respectively. The structural integrity of the material under full loading ensures its longer cyclic life as an anode. The respective values of open circuit voltage (OCV) are calculated as 3.31, 4.12, 1.09, 1.24, and 1.43 V for LIBs, KIBs, MIBs, CIBs, and AIBs, indicating the performance of LTO as an electrode in these batteries. Additionally, the migration paths of Li ions and vacancies were studied using the Cl-NEB , indicating low energy barriers and revealing the stability of the host. The minimum energy barriers faced by diffusing metal atoms are calculated as 0.52 eV (LIBs), 0.28 eV (KIBs), 0.43 eV (MIBs), 0.01 eV (AIBs), and 1.51 eV (CIBs). The vacancy migration pathways of the metal ions in the host material are also determined. Furthermore, the MD simulations at 300K to 900 K are studied to determine the diffusion coefficient and rate performance of LTO as an electrode, which appeared as 1.04 × 10^–12^ m^2^/s (LIBs), 0.83 × 10^–5^ m^2^/s (MIBs), 0.66 × 10^–9^ m^2^/s (AIBs), 0.07 × 10^–11^ m^2^/s (CIBs), and 7.65 × 10^–9^ m^2^/s (KIBs), respectively. The ionic conductivity of LIBs, MIBs, CIBs, KIBs, and AIBs appeared as 2.32 × 10^–3^ Sm^–1^, 1.19 × 10^–2^ Sm^–1^, 8.32 × 10^–2^ Sm^–1^, 6.33 × 10^–3^ Sm^–1^, and 0.21 × 10^–2^ Sm^–1^, respectively. The calculated properties point to the suitability of LTO as a promising electrode material in these batteries. Furthermore, to model the formation of the solid electrolyte interphase, the nonequilibrium Green’s function technique was used to study the transportation of electrons and current–voltage characteristics. The findings of the study suggest that LTO is a potential candidate for use as an anode in multivalent metal-ion batteries.

## Graphical abstract

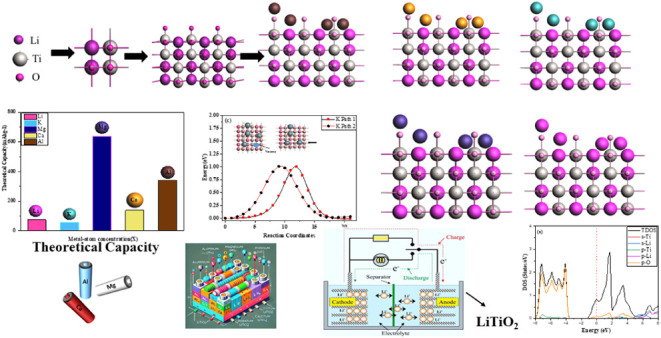

## 1 Introduction

The global energy demand is mostly met by fossil fuel-based nonrenewable energy sources.^1^ The depletion of these sources motivates researchers to develop affordable and sustainable power sources by harvesting geothermal, solar, wind, and tidal energy.^2^ The development of energy storage mechanisms is essential for integrating these sources into grids and ensuring a sustainable electrical energy supply to power devices and vehicles.^3^ The current technologies of storage devices, including electrochemical batteries, supercapacitors, lead–acid batteries, and chemical, mechanical, and thermal storage elements, suffers from issues such as poor energy density, volume expansion, cost-effectiveness, and environmental hazards.^4,5^ The performance of electrochemical storage is critically dependent on the electrode materials, which directly influence the efficiency, cyclic life, and overall viability of the device. In multivalent metal-ion batteries, the reversible transfer of metal ions takes place through the electrolyte in such a way that the ions released from the cathode intercalate into the anode during charging and deintercalate back during discharging.^6,7^ Earlier battery technology relied on water-based electrolytes, which have now been replaced by alternative liquid and solid electrolytes.^3^

The technological improvements in Li-ion batteries (LIBs) are driven by their superior practicality in advanced power systems, plug-in hybrid vehicles, and household gadgets. The utilization of lithium metal as an anode in rechargeable LIBs may be beneficial due to its low standard potential, high specific capacity, and low redox potential, but it suffers from challenges related to Coulombic efficiency, cyclic life, and anodic dendritic formation. Hence, based on the utilization of resourceful lithium compounds as anode materials, LIBs have witnessed exceptional research progress and industrialization in the recent past. It appears that LIB technology has reached a plateau in several areas, including pricing, safety concerns, and energy density issues. Research efforts to overcome these drawbacks are still in progress, thereby synthesizing novel lithium compounds. In this regard, the current work is carried out to model the operation of multivalent metal-ion batteries based on mono- and multivalent metal-intercalated lithium titanate oxide as electrodes. Metals with high charge capacity and multiple storage capabilities, such as magnesium, zinc, and aluminum, are used as anode materials in rechargeable batteries to enhance energy density.

The multivalent cations provide two or three electrons per ion, offering greater capacity when intercalated compared with monovalent lithium ions, assuming comparable amounts of intercalant sites are present in cathode materials. Though magnesium ions possess a similar ionic radius in comparison with that of lithium ions, they experience strong polarization-induced limitations and thus slow ion transport.^4^ The energy density and safety problems with batteries can be efficiently solved by using trivalent Al ions in AIB technology. The rechargeable AIBs form high-performance multivalent battery systems due to the trivalent character of Al ions, offering lower cost and high energy density when compared with other metal-ion batteries.^5^ Research efforts are in progress to enhance the technology of MIBs and AIBs, thereby searching for novel electrode materials to promote ionic transport, cyclic stability, and energy density.^6^

The anode materials used in current multivalent batteries are still far from being industrialized due to issues such as low-rate performance, slow ion mobility, and poor stability over charge/discharge cycles. To address these problems, layered materials are considered a viable solution for use as anodes in multivalent batteries.^7^ An anode material with medium voltage, when paired with cathodes, can be used in multivalent-ion batteries to deliver high capacity and, thus, elevated energy density at an affordable cost.

Despite the commercialization of LIBs for practical uses, considering their widespread applicability and future prospects, efforts to improve their performance are still in progress with an emphasis on the fabrication of novel electrode materials. The utilization of anode materials is not limited to LIBs alone but may be extended to any multivalent ion battery, provided that they can reversibly host metal ions (e.g., Mg^2+^, Zn^2+^, Ca^2+^, and Al^3+^, etc.) during intercalation/deintercalation. The utilization of the same anode material for multivalent-ion batteries should be tested, as it may provide opportunities to offer greater storage capacities when the intercalating ion can transfer two or more electrons.^8^ The most favourable energy sites for metal atoms in LTO structure are given in Table 1, and LiTiO_2_ (referred to as LTO) is a potential candidate for utilization in multivalent-ion batteries. The findings of the study demonstrate that the proposed compound offers good structural stability, suitable electronic properties, a low diffusion barrier, and favorable anodic properties, which predict its suitability for use as an anode in metal-ion batteries. The choice of LiTiO_2_ as a material in energy storage research, especially for multivalent-ion batteries (MIBs), is justified by its structural and electrochemical attributes. LiTiO_2_, particularly in its layered or spinel form, has a stable structure with low dimensionality; lithium-ion diffusion pathways and electronic conduction properties are achievable through modification or doping. Its theoretical capacity (∼165 mAh/g) and low volume change during cycling make it a promising candidate, suggesting better safety than graphite. In addition, titanium-based oxides are environmentally benign, inexpensive, and abundant. The Ti^4+^/Ti^3+^ composition provides feedback for reversible electrochemical reactivity, ensuring cycle life and stability at higher rates. It can provide solutions to overcome some of the main challenges associated with MIBs, such as safety, rate capability, and structural deformity.^5,9^

**Table 1:** Calculated Values of Energy for the High- Symmetry Interstitial Sites for Li, Mg, Ca, K, and Al Adsorption in LiTiO_2_

Interstitial sites in the host LiTiO_2_	Li Adsorption energy (eV)	Mg Adsorption energy (eV)	Ca Adsorption energy (eV)	K Adsorption energy (eV)	Al Adsorption energy (eV)
$B_{O}$	–3.33	–2.18	–4.43	–2.48	–12.89
$B_{\mathrm{Li}}$	–2.37	–1.45	–3.02	–1.39	–4.62
$B_{\mathrm{Ti}}$	–2.03	–1.06	–2.13	–0.22	–2.53
**B_Li–Ti_**	0.98	0.40	10.32	1.2	0.64

LiTiO_2_ is shown as a promising anode material because of its favorable electrochemical properties, including good structural stability, high theoretical capacity, chemical compatibility, and low diffusion barriers with both monovalent (Li^+^) and multivalent (Mg^2+^, Ca^2+^, Al^3+^) ions. In addition, its layered structure facilitates efficient ion intercalation/deintercalation, which is important for achieving high performance in next-generation multivalent metal-ion batteries (MIBs). The presence of titanium provides redox-active sites while maintaining a relatively low operating voltage, which is required for high energy density and safe battery operation.

## 2 Methodology

This work comprises density functional theory (DFT)-based calculations conducted using different modules of the ADF code, which is based on the linear combination of atomic orbitals. The quasi-Newton method was used to optimize the structures, with a step size of 0.001 and a convergence criterion around 10^–5^ eV/atom during SCF cycles. The electronic configurations of Li, Ti, and O are, respectively, (1s^2^, 2s^1^) and ([Ar] 3d^2^, 4s^2^) and (1s^2^ 2s^2^ 2p^4^) for which all-electron calculations were performed. The density-functional perturbation theory-based phonon calculations were carried out to predict the lattice modes and dynamical stability.^10^ High numerical accuracy was maintained by using the Becke fuzzy cell integration scheme while also ensuring a balance between computational efficiency and cost through the frozen core approximation. The ADF framework’s frozen core approximation was utilized to handle core electrons, while all-electron-like Slater-type orbitals were utilized to explicitly treat the valence electrons of Li, Ti, and O. The Self-Consistent Field (SCF) calculations were carried out using the Direct Inversion of the Iterative Subspace (DIIS) method, which applies a detailed integration approach to accurately evaluate the Hamiltonian and maintain reliable performance throughout the process. Single-point calculations were used to compute nuclear and Hessian gradients at specific positions to determine the site energies and electronic properties. For an accurate evaluation of site energies, separate calculations were performed for each specific site. To simulate the intercalation process, we calculated the intercalation energy at different symmetric positions by monitoring the exothermic nature of the reaction via negative values of adsorption energies. By identifying the location within the host material with the lowest (most negative) intercalation energy, we found the most favorable site for intercalation. The adsorption energies of X atoms (Li, Al, Mg, Ca, and K) in host LiTiO_2_ were calculated using eq 1.

1 $$E_{\mathrm{ads}}=E_{{Xn\mathrm{LiTiO}}_{2}}-n\times E_{X}-E_{{\mathrm{LiTiO}}_{2}}$$

where “*n*” is number of adsorbed atoms on the host structure, *E_x_* represents the adsorption energy of a single isolated metal atom *X* in its standard state, E_XnLiTiO2_ represents the intercalation energy of *X* atom (*X* = Li, Mg, Al, K, Ca) in LiTiO_2_, and E_LiTiO2_ is the energy of the pure LiTiO_2_ structure. The periodicity parameter was set to “slab”. The “GFN1-xTB” model is used, which divides molecules into fragments, enabling efficient calculation. The exchange correlation is taken as the Generalized Gradient Approximation (GGA-PBE) by using dispersion correction at Grimme’s D3 level of theory (DFT-D3). The ADF library’s Slater-type orbitals with “triple-ζ polarization” (TZP) were used to accurately represent the density near the nucleus. Molecular dynamics (MD) simulation was performed to calculate the thermodynamic properties of the structure using the ReaxFF module. To calculate the electrochemical performance of LiTiO_2_ serving as an anode material, the storage capacity was calculated using eq 2.

2 $$C_{M}=\frac{n\;*\;z\;*\;F}{M_{{\mathrm{LiTiO}}_{2}}}$$

Here, *F* represents Faraday’s constant, *n* represents the number of Li atoms involved in emulations, *z* indicates the outermost number (*z* = 1 for Li), and *M*_LiTiO2_ denotes the mass of LiTiO_2_. The open-circuit voltage (OCV) provides primary information for evaluating the electrochemical performance of any anode material, the value of which is calculated using a reversible voltage (eq 3).

3 $$\mathrm{OCV}\approx \frac{E_{{Xn\mathrm{LiTiO}}_{2}}-E_{X_{n1}{\mathrm{LiTiO}}_{2}}-\left(n_{2}-n_{1}\right)\mu_{X}}{z\left(n_{2}-n_{1}\right)e}$$

where $E_{X_{n2}{\mathrm{LiTiO}}_{2}}$ and $E_{X_{n1}{\mathrm{LiTiO}}_{2}}$ are the energy values associated with the intercalated host material having respective *n*_2_ and *n*_1_ concentration of *X* atoms, *μ_X_*, *e* and *z* denote the chemical potential of the guest atoms, electronic charge, and the oxidation state (*z* = 1 for Li, K; *z* = 2 for Mg, Ca; and *z* = 3 for Al), respectively.^11^

The process of intercalation of metal atoms can be examined by modeling their movement inside the host structure via different paths. The energy barriers faced by the intercalating atoms in the host structure were computed by using nudged elastic band (NEB) simulations to model the diffusion process.^12^ In this regard, the value of the energy barrier for the diffusion of *X* atoms (where *X* = Li, Mg, Al, Ca, or K) in the host LiTiO_2_ is calculated via the NEB methodology. The energy required by *X* diffusing atoms across the layers of the host structure helps to evaluate the charging/discharging mechanism of metal-ion batteries.^13^ During the Cl-NEB simulation, the spring constant is set at 1 Ha/Bohr^2^ with a skewness value of 1. The energy criteria are set at 10^–5^ eV with a step convergence of 0.01 Å, while the restart displacement is 0.05 Å. The magnitude of the force acting on the analyzed representation is calculated using eq 4.

4 $$F_{i}=\left|F^{s}{|}_{||}-\right|\nabla E\left(R_{i}\right){|}_{⊥}$$

where *F_i_* indicates the overall force on the *i*th picture, *F_i_^s^*_||_ represents the spring force, and |∇*E* (*R_i_*)|_⊥_ shows the actual force.^12^ The state with feasible energy is produced by using eq 4.

In order to determine the diffusion coefficient, MD simulations are utilized while incorporating the Einstein relation, according to which the self-diffusion coefficient of any atom moving randomly in three dimensions is determined by plotting the limiting slope of the mean-square displacement (MSD) vs time.^14^ Equations 5 and 6 are used to determine MSD and the diffusion coefficient, respectively.

5 Mean Square Displacement=⟨|r(t)−r(0)2|⟩

6 $$\mathrm{Diffusion coefficient}=\frac{1}{6N}\underset{t⃗\infty }{\lim}\frac{d}{dt}\overset{N}{\underset{t=1}{\sum }}\left\langle \right|r_{i}\left(t\right)-r_{i}\left(0\right){|}^{2}\rangle$$

where the position vector of the atom at time *t* and *t* = 0 is indicated by the symbols *r*(*t*) and *r*(0), respectively. The ionic conductivity is determined by eq 7:

7 $$\sigma =\frac{nq^{2}}{K_{b}T}D$$

where “*T*” is the temperature in ionic conductance, “*q*” is the amount of charge, ″*D*” is the diffusion rate coefficient, and *K_b_* is the Boltzmann constant. Here, “*n*” denotes the ionic carrier concentration (number of mobile charge carriers per unit volume), which is the quantity of mobile Li^+^ ions per unit volume that contribute to ionic conduction. This is different from eq 1 which denotes the number of adsorbed/intercalated atoms on the host structure.

The NEGF computation was implemented to find the current–voltage (*I–V*) characteristics to understand the formation of the SEI.^15^ The NEGF provides information on electron transport and uses the Landauer-Büttiker formula, given in eq 8, to obtain the current, *I*.

8 $$I\left(V\right)=2e/h\int T\;\left(E,V\right)\left(f\left(E-\mu_{L}\right)-f\left(E-\mu_{R}\right)\right)dE$$

## 3 Results and Discussion

The results calculated on the structural and electronic properties, thermal stability, metal atom storage capacity, and transport properties of LiTiO_2_ for application in Li-, Na-, Mg-, Ca-, and K-ion batteries are described in the following sections.

### 3.1 Structural Properties

The various structural forms of LiTiO_2_ are primarily crystallized in different forms depending on synthesis conditions, with the rock salt-type cubic as the most stable structure, having a lattice parameter of 4.1 Å with the space group Fm3m. The structure possesses a disordered cation arrangement, considering the random distribution of lithium (Li) and titanium (Ti) ions over octahedral sites within a face-centered cubic (FCC) oxygen sublattice, resulting in a dense and sturdy structure. This phase is thermally resistant because of its formation under high-temperature conditions via solid-state reactions.^16^ In addition, LiTiO_2_ can also crystallize in a deformed perovskite-like configuration with the space group Pbnm, forming an orthorhombic layered structure where TiO_6_ octahedra create a layered framework with well-defined planes. Potential ion diffusion paths with specific crystallographic directions are possible due to the presence of lithium ions in the interlayer gaps. In comparison with the rock-salt phase, this layered arrangement increases lithium mobility, making the orthorhombic form advantageous for electrochemical applications, specifically in LIBs.^17^ The spinal LiTiO_2_ is a metastable phase that crystallizes in the cubic spinel structure, where Li ions are partially dispersed throughout the tetrahedral sites and Ti ions occupy the octahedral sites. The spinel structure is beneficial for electrochemical applications like LIBs due to the interconnected channels generated by its three-dimensional layout, which may help in lithium-ion diffusion.^18^

When a Li^+^ ion was bonded to six O^2–^ ions, it formed a LiO_6_ octahedron. This LiO_6_ octahedron is connected in such a way that it shares a corner with two TiO_6_ octahedra, shares corners with four other LiO_6_ octahedra, shares edges with four LiO_6_ octahedra, and shares edges with eight TiO_6_ octahedra.^19^ Similarly, when a Ti^3+^ ion bonds with six O^2–^ ions, it forms a TiO_6_ octahedron. This TiO_6_ octahedron shares corners with two LiO_6_ octahedra, shares a corner with four TiO_6_ octahedra, shares edges with four TiO_6_ octahedra, and shares edges with eight LiO_6_ octahedra. This structure helps form a stable and interconnected crystal network, necessary for ion movement in battery materials. The structure comprises two longer (2.10 Å) and four shorter (2.02 Å) Ti–O bonds.^20^ The tetragonal phase of LiTiO_2_ is used because of its unique properties, such as layered structures,^21^ which favor ion intercalation, a low diffusion barrier, and better rate capability. Most importantly, it shows great structural stability,^22^ which allows minimal volume change during charging and discharging. It also shows high compatibility with multivalent ions and provides favorable electronic properties.^23^ The unit cell of bulk LiTiO_2_ , consisting of 16 atoms, is shown in Figure 1a,b. The calculated values of the lattice constant of the LiTiO_2_ unit cell are *a* = *b* = 4.04 Å and *c* = 8.41 Å, with the volume as 137.8 Å³, which accords with the relevant literature.^24^ The bond lengths of the LiTiO_2_ unit cell for Ti–O and Li–O were found to be 2.02 Å, and those for Li–Ti and O–O were found to be 2.91 Å. The dihedral bond angles O–Li–O, O–Ti–O, and O–Li–Ti for the unit cell appeared as 90°, 90°, and 46°.^25^ In order to investigate the intercalation process in the material, a 3 × 3 supercell having the formula unit Li_36_Ti_36_O_72_ comprising 144 atoms, was optimized with the lattice parameter as *a* = *b* = 12.13 Å. The value of the formation energy was calculated by using a formula (eq 9).

9 $$E_{f=}E_{\mathrm{System}}-\sum E\left(\mathrm{atomic}\right)$$

**Figure 1:**
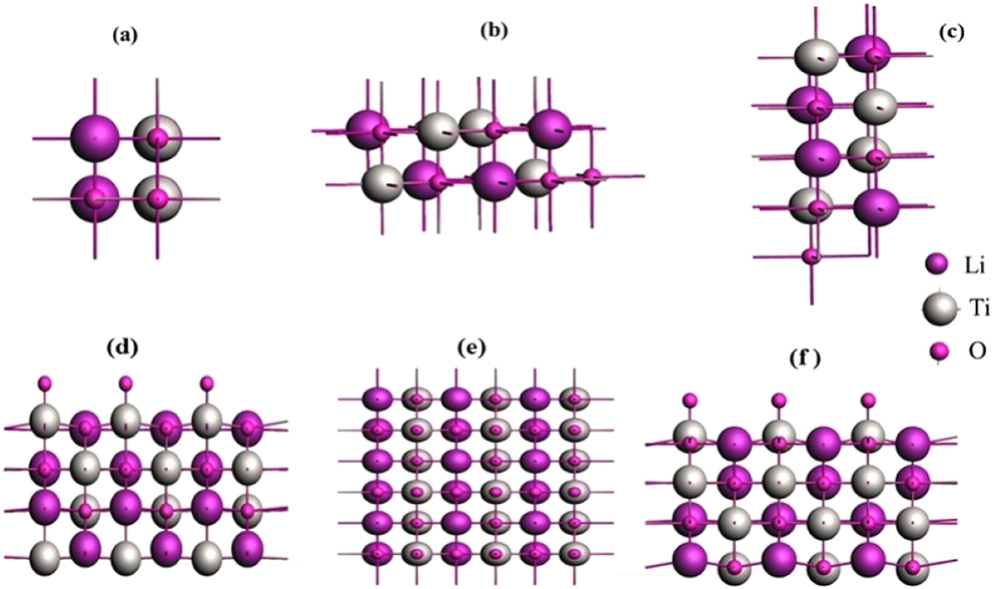
Optimized structure of LiTiO_2_ of unit cell: (a) along the *x*-axis, (b) *y*-axis, and (c) *z*-axis. The optimized 3 × 3 supercell slab (d) along the *x*-axis, (e) *y*-axis, and (f) *z*-axis.

The formation energy for the unit cell was calculated as −2.97 eV, which appeared as −4.32 eV in the case of the supercell of the material.^26^ The findings revealed a stable structure of the material formed under exothermic conditions.^26–28^ The total energies of the primitive unit cell and the supercell are −2.97 eV and −4.32 eV, respectively, which are reported formation energies. The first version did not normalize these values. We have now normalized and recalculated the formation energies per formula unit (f.u.) to ensure clarity and meaningful comparison. The internal unity of the computations is demonstrated by the consistent formation energy values per formula unit that both the supercell and unit cell produce after normalization.

### 3.2 Electronic Properties

The electronic configuration of the constituent elements contributes to the formation of bonds, resulting in the unique structural and chemical properties of LiTiO_2_. The electronic properties of the material were investigated in order to explore its suitability for utilization as an anode in multivalent-ion batteries. The density of states (DOS) and band diagram calculated for the material are shown in Figure 2 which shows the metallic nature of the material, in agreement with the literature.^27^ LiTiO_2_’s density of states (DOS) and band structure demonstrate metallic behavior, showing excellent electronic conductivity that facilitates successful charge transport in the battery electrode. In multivalent-ion batteries, where sluggish ion diffusion often restricts performance, this property becomes even more essential. The mobility of multivalent ions may be enhanced by quicker electron transfer made possible by the electronic delocalization induced by Ti-3d and O-2p hybridization.^28^ The valence electronic configuration of Li (1s^2^) and Ti (3p^6^4s^2^) indicates the involvement of Ti-3d and Ti-4s unpaired electrons in the bond formation of the material. One unpaired electron in the Li-2s orbital, due to valence configurations 1s^2^2s^1^ is involved in the formation of bonds. Figure 2b shows the TDOS of LiTiO_2_ together with the partial DOS related to Ti, Li, and O, which points to the hybridization of Ti-4s and Ti-3d orbitals with the O- and Li-related states to exhibit bond formation. Ti forms multiple bonds with Li and O in the form of single bonds, contributing to the stable structure of the material. The configuration of O (2s^2^2p^4^) points to the formation of two bonds due to two unpaired electrons, enabling orbital hybridization in LiTiO_2_ to establish a maximum of two bonds. Hence, the material exhibits a unique bonding configuration where Ti and O form a covalent bond, while Li and Ti facilitate metallic character.

**Figure 2:**
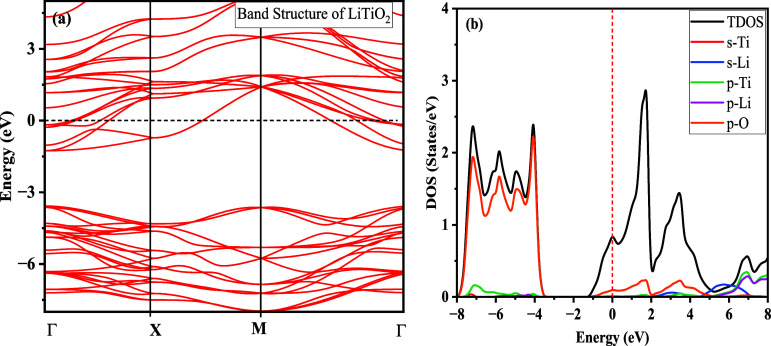
Calculated electronic properties of LiTiO_2_ in the form of: (a) band structure and (b) density of states (DOS). The Fermi level is adjusted to 0 eV.

The structural durability of LiTiO_2_ can be attributed to the presence of a strong covalent link between Ti and O, in addition to the metallic interactions involving Li. For long-term cycle durability in electrochemical settings, such a stable bonding framework is highly beneficial.^29^ The O-p orbitals and Ti-3d orbitals play an important role in the electrical conductivity of the material. The dominant role of O-related states is found near the Fermi level, whereas the involvement of the Ti-p orbitals is found away from the Fermi level. LiTiO_2_ displays robust electronic properties, favorable for metal-ion batteries, as compared to traditional Ti-based anode materials like TiO_2_ or Li_4_Ti_5_O_12_. It may contribute to enhanced rate capability and electrical conductivity, which are crucial features required for high-performance multivalent batteries.^30^

### 3.3 Favorable Adsorption Sites

In order to know the suitable intercalation site, we carried out energy profiling of Na, Mg, Ca, K, and Li at different sites of the host structure LiTiO_2_. The concentration of the foreign atoms was gradually increased to determine the adsorption energy using eq 1. Figure S8 shows four high-symmetry locations for Li, K, Mg, Ca, and Al adsorption in the host material. The first high-symmetry site is B_O_ which is located close to the O atom, and the second site is B_Li_ which is positioned near the Li atom of either layer (upper). B_Ti_ is the third site, which is located at the Ti atoms of the upper and bottom layers, whereas the fourth high-symmetry site is B_Ti–Li_ which is situated near the middle of the Ti atom of one layer and the Li atom of the other layer. The favorable intercalation sites for Li are illustrated in Figure S8d. The BO site is the most suitable site for Li adsorption, with an energy of −3.33 eV, which is owing to the high value of electron affinity of the O atoms in the structure. This preference is due to to the strong electrostatic interactions between the intercalating cation (Li^+^) and the nearby oxygen atoms, which have high electronegativity and create a favorable local electronic environment. The oxygen-rich region at the BO site increases charge transfer and binding strength, making it energetically the most favorable site for Li adsorption. The second most favorable adsorption site is B_Ti_, with an intercalation value of −2.37 eV. The third most supportive site is B_Li_, which yielded an adsorption energy of −2.03 eV. The site B_Ti–Li_ corresponds to 0.98 eV, pointing out its unsuitability for adsorption due to the small value of electron affinity of Ti and Li in the base material. Correspondingly, the suitable adsorption sites for Mg, Ca, K, and Al are shown in Figure S8, which follow the same trend as observed in the case of Li. The size and charge of the intercalating species, together with the electronic configuration and site proximity, help to understand the variation in adsorption energies. Due to their larger charge densities, Mg^2+^ and Al^3+^ for instance, have stronger electrostatic interactions with oxygen-rich sites like B_O_. Yet, they may also undergo more repulsive strain in less electronegative environments like B_Ti–Li_, which could lead to poor adsorption stability.^31,32^ It is interesting to analyze that a comparable site preference pattern has been reported in Li_4_Ti_5_O_12_, where strong Coulombic interactions enable oxygen-rich sites to offer the most stable adsorption energy for Li^+^ and Mg^2+^.^33^ Fortunately, due to their divalent nature and site distortion, Mg and Ca commonly face greater migration barriers in spinel-type oxides, emphasizing LiTiO_2_’s beneficial lattice compatibility.

### 3.4 Electronic Properties of Li-, Mg-, Ca-, K-, and Al-Adsorbed LiTiO_2_

The analysis of the DOS of the Li-, Mg-, Al-, K-, and Ca-intercalated LiTiO_2_ is carried out to investigate the host’s chemical reaction with the adsorbed atoms. Figure S1a shows the DOS of the Li-adsorbed structure, calculated to explore the intercalant’s engagement and the influence of electron transport from Li to the host. The DOS chart of the Mg-adsorbed host structure is illustrated in Figure S1b in which the orbital hybridization sheds light on the transfer of electronic charge from Mg valence states to the host structure. The comparison of different cases indicates that Al can easily release electrons due to a high density of states near the Fermi level, as per Figure S1c. The high density of occupied states related to the intercalant in Al: LiTiO_2_ facilitates the transfer of Al-3p electrons to the conduction band of the host. In the same view, Ca and K atoms donate their valence electrons to the host easily due to the availability of high DOS at the Fermi level, as per Figure S1d,e. The analysis of the DOS helped to understand the interactions between the host material and the electronic states of the intercalants in Mg-, Li-, K-, Al-, and Ca-intercalated LiTiO_2_. Figure S1c sheds light on electronic charge transfer via Al’s high DOS near the Fermi level, similar to that of LiCoO_2_, where the existence of a high density of electronic states close to the Fermi level enables Li^+^ intercalation at higher rates.^33^ LiTiO_2_ intercalant behavior is similar to that of other Ti-based anodes, such as Li_4_Ti_5_O_12_. The orbital hybridization demonstrated in Mg-intercalated LiTiO_2_ (Figure S1b) contrasts with what is observed for Li^+^ and Mg^2+^ ions in Li_4_Ti_5_O_12_, where the electronic conductivity and rate performance are significantly affected by the interaction between the intercalants and the host structure.

LiTiO_2_ and Li_4_Ti_5_O_12_ are both anodes, but LiTiO_2_ shows a higher density of states (DOS) close to the Fermi level when intercalating with multivalent ions such as Mg^2+^, Al^3+^, and Ca^2+^. This leads to stronger orbital hybridization between the intercalant valence orbitals (such as Mg-3s, Al-3p) and the O-2p/Ti-3d orbitals of the host lattice. The interaction shows efficient charge transfer and enhanced electronic conductivity. On the other hand, for divalent cations, e.g., Mg^2+^, Li_4_Ti_5_O_12_ shows weaker orbital overlap close to the Fermi level, which results in lower electronic conductivity and limited rate capacity. For large or multivalent ions, the comparatively lower DOS in Li_4_Ti_5_O_12_ near the Fermi level leads to decreased intercalation kinetics and worse electron mobility. This difference explains why LiTiO_2_ is a better anode for improved electrochemical performance and multivalent ion compatibility, making it a more appropriate anode for advanced battery applications. However, in comparison with LiTiO_2_, the structure Li_4_Ti_5_O_12_ exhibits a reduced DOS close to the Fermi level, which results in poor electron transport, particularly for larger or divalent ions like Mg^2+^.^29^ Both Ca^2+^ and K^+^ in LiTiO_2_ (Figure S1d,e) smoothly donate their valence electrons to the host because of the high density of the occupied states at the Fermi level, which ensures electron transmission and enhances the ion insertion process. This is identical to what has been noticed in LiFePO_4_, where enhanced ion insertion and electron transfer are prompted by the high DOS for Li^+^.^34^ Mg^2+^, Al^3+^, Ca^2+^, and K^+^ exhibit orbital hybridization and electron transfer to the host structure in LiTiO_2_, suggesting that it could be a potential material for multivalent-ion batteries. Hence, in the range of LTO systems, LiTiO_2_ offers high DOS at the Fermi level, which improves electron mobility and ionic conductivity—vital elements for high-performance multivalent-ion batteries. However, due to the greater ion size restrictions and comparatively lower DOS, LiFePO_4_ and LiCoO_2_ show weaker compatibility with multivalent ions, despite having similarly favorable electrical properties for Li^+^ intercalation.^35^ Thus, the rate capability and electrochemical stability of LiTiO_2_ are shown to be superior for multivalent ion intercalation, specifically in high-performance battery applications, compared to other well-known materials like Li_4_Ti_5_O_12_ and LiCoO_2_ , taking into consideration electron donation from intercalants like Mg, K, Ca, and Al.

### 3.5 Dynamic and Thermal Stability

The atomic arrangement and the relevant force constants in the case of the monolayers are reflected in vibrations on their surface.^36^ The dynamic stability of LiTiO_2_ is studied by the calculation of phonon dispersion curves, as given in Figure 3a, which illustrates the calculated phonon band structure. The analysis of the results clearly shows a lack of imaginary modes, which points to the dynamic stability of the material. The material’s capability of handling the thermal stresses occurring during charge and discharge cycles points to the importance of dynamical stability in preserving its structural integrity during these cycles. The dynamical stability in titanium oxide polymorphs has shown the worth of these structures for such applications.^37^ The availability of higher DOS near the Fermi level is helpful in improving carrier conductivity and mobility, revealing that LiTiO_2_ stands out in comparison to conventional electrode materials like LiFePO_4_ and LiCoO_2_. However, despite having beneficial electronic characteristics for Li^+^ intercalation, LiFePO_4_ and LiCoO_2_ are less appropriate for application in multivalent-ion batteries due to their lower DOS and structural constraints.^20^

**Figure 3:**
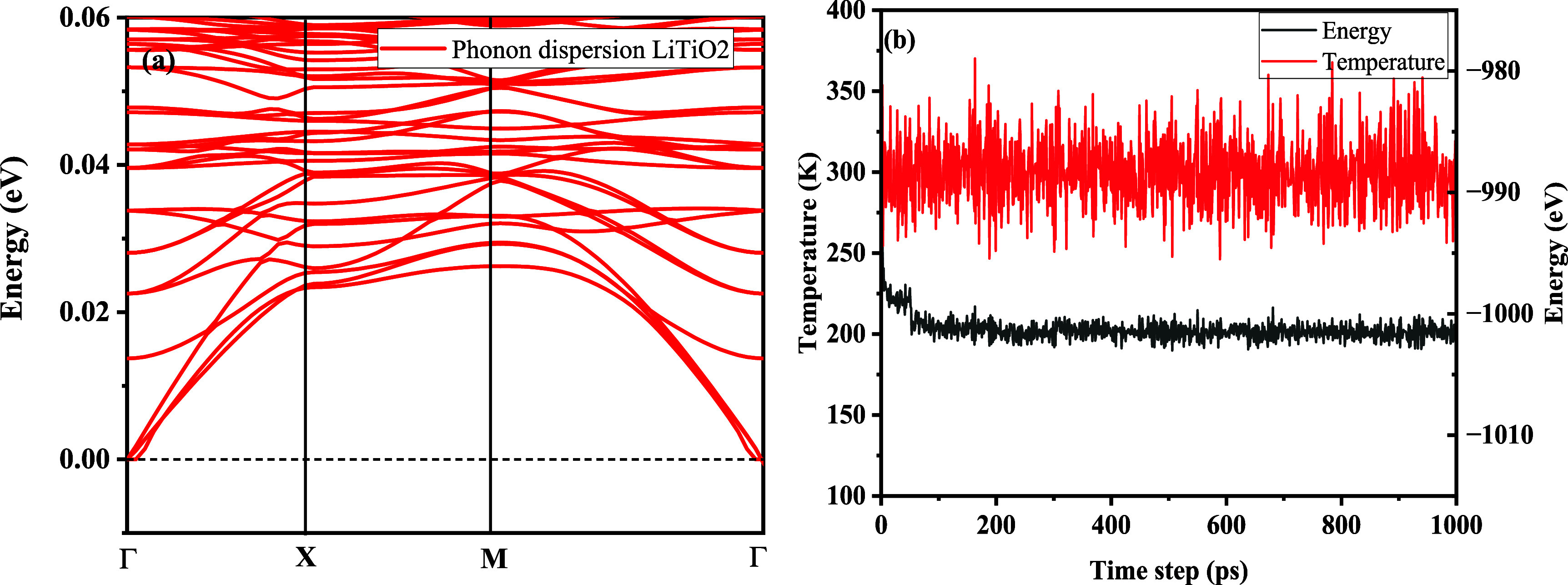
(a) The phonon band structure of LiTiO_2_ and (b) computed AIMD simulations illustrating how the energy changes with temperature after 1000 ps.

During battery operation, the generation of heat and overheating can lead to thermal runaway, making the analysis of the thermal properties of the anode material essential to investigate. In this regard, AIMD simulations were conducted on 3 × 3 × 1 supercell of the material at 900 K for 1000 ps with a time step of 0.5 fs to investigate the thermal stability of LiTiO_2_. Figure 3b shows the calculated temperature and energy profiles as a function of time. The observation of small fluctuations ensures that the structure retains its stability through thermal shocks during battery operation. On the contrary, studies on rutile TiO_2_ under high thermal pressure have shown soft phonon modes when evaluating thermal stability in battery applications.^38^ Furthermore, the AIMD simulation was applied to simulate the possible incorporation of 7 Li, 19 Mg, 5 K, 6 Ca, and 11 Al atoms (the maximum numbers obtained after ensuring exothermic conditions) in the host material. These calculations help in finding the optimal stoichiometric ratio required to maintain charge neutrality and structural stability, rather than relying solely on DFT-based energies to calculate the adsorption energy via eq 1. The adsorption of Li, Mg, K, Ca, and Al atoms in the host ensures intercalation without any sign of metallic clustering or dendritic development. This behavior is in accordance with the outcomes of previous studies that have shown that adding Li, Al, and Mg to rutile VO_2_ promotes faster ion diffusion and higher voltages, suggesting the ideal conditions for multivalent ion intercalation.^20^ The stability of energy and temperature of the fully loaded structure over time during AIMD simulations at 900 K for LIBs, MIBs, CIBs, KIBs, and AIBs, respectively, is shown in Figure 6.

### 3.6 Adsorption Energy Calculations

The sequential insertion of metallic elements into the host LTO plays an important role in modeling the charge/discharge cycles of batteries.^39^ The addition of successively increasing numbers of Li, Mg, K, Ca, and Al into the host was carried out, thereby monitoring the exothermicity of the reactions. In the case of Li adsorption, it appears that the reaction remained exothermic until the seventh atom was added to the host. However, the reaction turned endothermic upon the adsorption of the eighth and subsequent Li atoms into the host, which points to an unfavorable reaction afterward, as shown in Figure 4a. The pictorial view of Li atoms adsorbed in the host, viewed from different directions, is given in Figure S9a.

**Figure 4:**
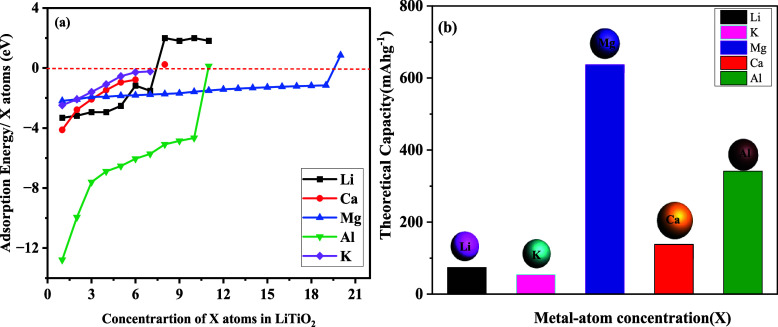
Computed values of (a) adsorption energy and (b) capacity of *X* atoms (*X* = Li, K, Mg, Ca, Al) as a function of concentration of Li, K, Mg, Ca, and Al atoms.

The second Mg atoms were placed on various sites of the optimized host structure to determine the adsorption energies. Adsorption energy was calculated by using eq 1, as described before. Until the 19th atom, the energy was negative, indicating an exothermic reaction, and the energies were favorable. After that, for the 20th atom, the energy became positive, leading to an endothermic reaction, which means the energies were not favorable now, so we stopped adsorbing atoms, as evidenced in Figure 4a. Three Mg atom layers formed on the optimized host structure LiTiO_2_, which can be shown in Figure S9d. Similarly, studies on layered Ni_0.75_O_2_ have demonstrated favorable adsorption energies for Mg ions, showing its potential for multivalent-ion batteries.^40^ Afterward, the adsorption energy of Al for the host structure LiTiO_2_ was calculated by placing an Al atom on the optimized host structure. It appeared that the adsorption energy remained favorable until the placement of the 11th atom, after which the reaction became endothermic, indicating unfavorable conditions. Figure 4a shows the adsorption of Al on the LiTiO_2_ structure; in Figure S9e, all the sides of adsorbed atoms of Al are shown. In the next phase, K atoms were added to the base structure, and the adsorption energy was calculated by using the adsorption energy formula. The energy remained exothermic until the seventh atom; after this, the energy became endothermic, as shown in Figure 4a. This behavior shows the larger ionic radius of K^+^, which may have introduced spatial constraints within the LTO structure, restricting its intercalation capacity. Likewise, in materials like LiCoO_2_, the insertion of larger alkali ions, such as K^+^, is obstructed by size mismatches and structural incompatibilities.^41^ Lastly, Ca was placed in the structure to determine the adsorption energy. Six atoms were adsorbed in the optimized host structure under an exothermic reaction, as evidenced in Figures 4a and S9b has shows the structure after adsorption, with all three sides visible. The type of reaction during the intercalation process (exothermic or endothermic) plays a critical role in reorganizing the behavior of the anode material. The outcome reveals an exothermic reaction, as the value of open circuit voltage of the host material for metal atoms shown in Figure 5.The spike in the concentration of Li/Mg/Al/Ca/K atoms within the host matrix was studied, and the energy released, associated with the electron transfer process, was computed using eq 1. The onset of negative to positive energy values is usually needed to calculate the storage capacity. The practical storage capacity considers the voltage profile to be a crucial factor. The material’s maximum storage capacity is considered only at the saturation value of Li, Mg, or Al concentration, where there is either a positive voltage or an absence of visible clustering.^42^

**Figure 5:**
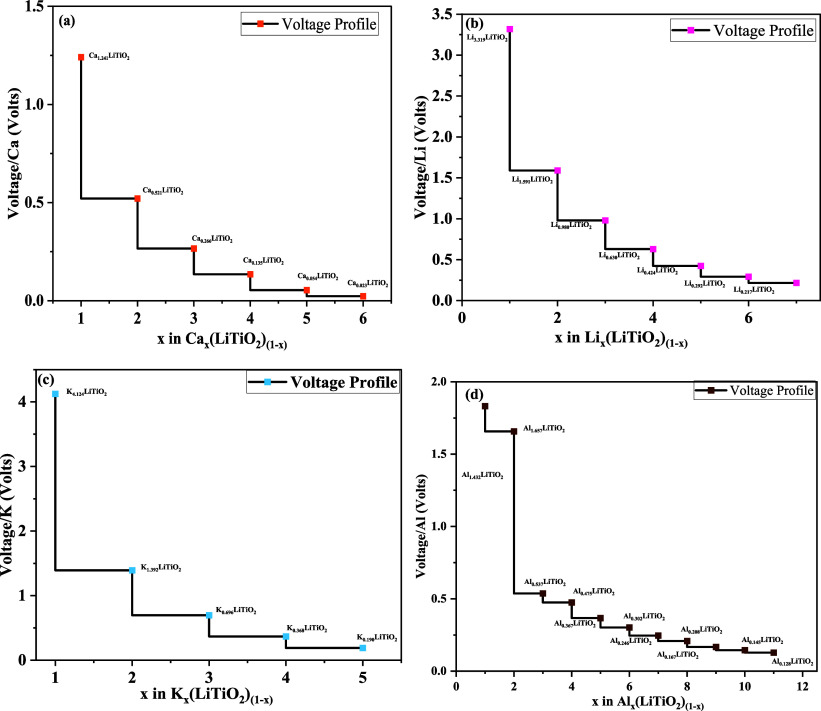
Voltage profile calculated for LiTiO_2_ showing voltage as a function of metal-atom concentration in volts in the case of (a) Ca, (b) Li, (c) K, and (d) Al.

### 3.7 Storage Capacity

The storage capacity of a material for utilization as an electrode material is of prime importance in predicting its performance.^43^ Its value for transition metal oxides can be evaluated using eq 2 for application as an anode material in batteries at the relevant chemical reactions.^44^ The storage capacity of the host material LiTiO_2_ as an anode material in AIBs, is calculated by monitoring the exothermic condition of the reaction until the adsorption of the 11th atom. The storage capacity of Al is calculated as 1131 mAhg^–1^ as illustrated in Figure 4b. A redox polymer capable of inversely inserting two anions (AlCl_4_) has been used to achieve a capacity higher than that of graphite.^45^ The storage capacity is comparable to that of NbS_2_-based anodes, which have demonstrated a value of about 1130 mAhg^–1^.^46^ The storage capacity of the host for MIBs, determined in the case of exothermic adsorption for the 19th Mg atom, resulted in 1302 mAhg^–1^, as shown in Figure 4b.^47^ The computed storage capacity of the host for CIBs is calculated as 411 mAhg^–1^ for six adsorbed Ca atoms, as illustrated in Figure 4b, which is comparable to several reported anodic materials. For example, CO_9_S_8_ anode has a reversible capacity of 500 mAhg^–1^.^48^ It offers superior performance in some cases and is competitive with several reported anode materials.^49^ The calculation of the storage capacity in the case of K indicates a value 171 mAh^– 1^, as illustrated in Figure 4b, which is lower compared to other materials such as δ-K_*x*_V_2_O_5_.*n*H_2_O which shows an initial capacity of 226 mAh^–1^.^50^ At last, the value of the capacity calculated in the case of LIBs appeared as 239 mAhg^–1^,^39^ which is on the same level as other titanium-based anodes, such as TiO_2_ nanotubes, which display capacities of nearly 245 mAhg^–1^ after 100 cycles.^51^ These findings reveal the potential of LiTiO_2_ as a robust anode material that can be employed for different ions, especially Al^3+^ and Mg^2+^, with competitive capabilities.

### 3.8 Voltage Profile

The open-circuit voltage (OCV) is a key parameter for assessing the efficiency of multivalent-ion batteries. The calculation of the storage capacity via Equation 3 involved positive OCV values or the absence of clustering in the case of the studied adsorption of Li, Mg, Al, Ca, and K.^52,53^ It sheds light on the relevant electrochemical processes, which play an important role in assessing the performance of batteries.^54^ The calculated voltage profile helps in modeling the charging and discharging phases of battery operation.^55^ The adsorption energy values calculated for the respective metal atoms employed in the determination of the OCV are given in Table S1. The respective values of open-circuit voltage (OCV) are calculated as 3.31, 4.12, 1.09, 1.24, and 1.43 V for LIBs, KIBs, MIBs, CIBs, and AIBs, respectively, as shown in Figure 5a–d, whereas the Mg case is given in Figure S2. The pattern indicates that the OCV value decreases as the concentration of metallic elements in the host material increases. The calculated OCV values in the case of host LiTiO_2_ indicate the electrochemical feasibility of the host for its utilization as an anodic material in multivalent batteries.

LiTiO_2_ exhibits comparatively low OCV values when compared to other transition metal oxides. For example, the famous LIB anode material Li_4_Ti_5_O_12_ has a flat voltage plateau of approximately 1.55 V vs Li^+^/Li due to its spinel structure and two-phase reaction mechanism, which enables reversible lithiation/delithiation.^56^ Mg-intercalated Mo_6_S_8_ in the Chevrel phase exhibited an average voltage of about 1.1 V due to the strong Coulombic interactions, thus offering sluggish kinetics.^57^ In the same way, Al intercalation in V_2_O_5_ produces OCV values between 1.3 and 1.5 V, which are higher than those of LiTiO_2_ but are often accompanied by poor cycle stability due to slow ion diffusion and volume fluctuations. Likewise, intercalation into TiS_2_ and Prussian blue analogs has led to voltages of nearly 0.2 to 0.5 V in the case of Ca-ion batteries.^58^ Therefore, LiTiO_2_’s OCV values are smaller than those of several contemporary electrode materials, especially for multivalent-ion batteries, where structural compatibility and dendritic pattern suppression are more crucial than voltage alone. The parameters calculated for the anode material LiTiO_2_ and a comparison with the literature are given in Table 2.

**Table 2:** Parameters of the LiTiO_2_ Anode Material and Comparison with Literature

Anode materials	Capacity (mAh/g)	Open-circuit Voltage (V)	Energy barrier (eV)	Reference
LiTiO_2_ (for LIBs)	240	3.31	0.52	Current work*
LiTiO_2_ (for MIBs)	1302	1.09	0.43	Current work*
LiTiO_2_ (for AIBs)	1131	1.43	0.01	Current Work*
LiTiO_2_ (for KIBs)	171	4.12	0.28	Current Work*
LiTiO_2_ (for CIBs)	411	1.24	1.51	Current Work*
Green phosphorus (GP) (LIBs)	288.4	2.07	0.14	^59^
TiClO (MIBs)	1079	0.96	0.68	^60^
B_2_C (MIBs)	3187.55	0.46	0.91	^61^
MgCu_2_ (MIBs)	1688	0.221	0.307	^62^
2D Scilicether (MIBs)	744	0.84	0.21	^63^
AlP (LIBs)	924	0.56	0.38	^64^
Si_2_BN (LIBs)	647.896	0.6–0.7	0.08–0.35	^65^
TiB_4_ (LIBs)	2353.2	---	0.24	^66^
GeP_3_ (LIBs)	648	0.4	0.5	^67^
Covalent triazine framework (LIBs)	925.99	0.51	0.65	^68^
TiC_3_ (LIBs)	1916	0.26	0.25	^69^
WC_4_ (LIBs)	577	0.65	0.55	^70^

### 3.9 Diffusion Barrier and Vacancy Migration Pathways

The energy barrier faced by diffusing metal ions must be calculated in order to model the charge/discharge process in multivalent-ion batteries.^71^ In this regard, the Cl-NEB method is used to understand the diffusion mechanism of intercalants in the anode materials. The height of the transition barrier considerably influences the metal atoms passing through the host via different paths during the charge/discharge cycles of the battery. The migration paths calculated for the diffusion of Li, Mg, Ca, K, and Al in LiTiO_2_ in two different tracks by taking the middle image are calculated via Cl-NEB. The reactions of *X*_*x*_LiTiO_2_ cases from starting to transition and then to the last state are illustrated in Figure 6a. The transition state displaying the energy barrier faced by Li in the host structure is studied via modeling Li_*x*_LiTiO_2_ across the paths. As illustrated in Figure 6a, the first path is captured between the first Li and second TiO_2_ layers, beginning from the center point to the end position, with Li positioned between Li and TiO_2._ The gap between the second TiO_2_ and third Li layer gives access to the second migration path, as illustrated in Figure 6a, and Li is placed between the oxygen atoms. In the same way, the migration paths of Mg, Ca, K, and Al are calculated. The diffusion barriers of Li, K, Mg, Al, and Ca are calculated as 0.5, 0.3, 0.43, 0.01, and 1.5 eV, respectively, as represented in Figure 6a–d, whereas the Ca case is given in Figure S3. The comparative analysis points that Al offers the lowest diffusion barrier among the studied cases. In recognition of its outstanding structural stability and “zero-strain” behavior, Li_4_Ti_5_O_12_ is a famous anode material for LIBs. Depending on the computational technique and crystallographic direction, the reported diffusion barrier for Li in LTO varies between 0.3 and 0.5 eV. It shows similar diffusion performance to the 0.5 eV barrier observed for Li in LiTiO_2_.^72^ TiO_2_-based materials have been thoroughly explored for the storage of Li and multivalent cations, especially in the anatase form. Based on the reports, the diffusion barrier in anatase TiO_2_ for Mg^2+^ may exceed 1.2–2.0 eV, which restricts its practical usage. The value of 0.43 eV for Mg in LiTiO_2_, on the other hand, describes noticeably better ion mobility, possibly as a result of LiTiO_2_’s increased interlayer spacing and host structure flexibility.^73^ Due to its layered structure, V_2_O_5_ is known as a flexible host material for multivalent ions. Considering their size and high binding energy, the reported diffusion barriers for Ca are noticeably larger than those for Mg, which lies from 0.7 to 0.9 eV.^74^ This shows that LiTiO_2_ offers a more kinetically favorable environment for Mg transport than V_2_O_5_, with a 0.43 eV barrier for Mg in LiTiO_2_. The relevant research interest in MXenes, for example, TiC_2_, is growing due to their excellent conductivity and 2D layered structure. Based on particular theoretical studies, depending on surface termination, the Al^2+^ diffusion barrier in Ti-based MXene can be as low as 0.5–0.1 eV.^75^ The comparative analysis indicates that LiTiO_2_ is a promising host for Al-ion batteries due to its low barrier of 0.01 eV. The outermost electronic structure of the shell plays an important role in determining the ability of a material to accommodate structural changes. The variability in minimum diffusion energy barriers for metal ions can be associated with their ionic size, charge, charge concentration, and strength of interaction with the host. Larger ions (K^+^) have lower charge density, causing weaker electrostatic interactions with the host lattice and lower diffusion barriers (0.28 eV). Smaller or more highly charged ions (Ca^2+^: 1.51 eV and Mg^2+^: 0.43 eV,) correspond to stronger interactions and higher barriers.^5^ The significant drop in diffusion barrier for Al^3+^ (0.01 eV); however, this can be attributed to its small ionic radius (53.5 pm), high charge, strong electrostatic interactions with the host lattice, and being more polarizable to a larger degree. Polarizability can involve localized lattice relaxation or charge redistribution, resulting in a smoother potential energy surface, which facilitates migration. For some 2D or layered materials, Al^3+^ ions could be stable in multicenter and delocalized charge environments, possibly lowering the ion hopping barrier.^76^

**Figure 6:**
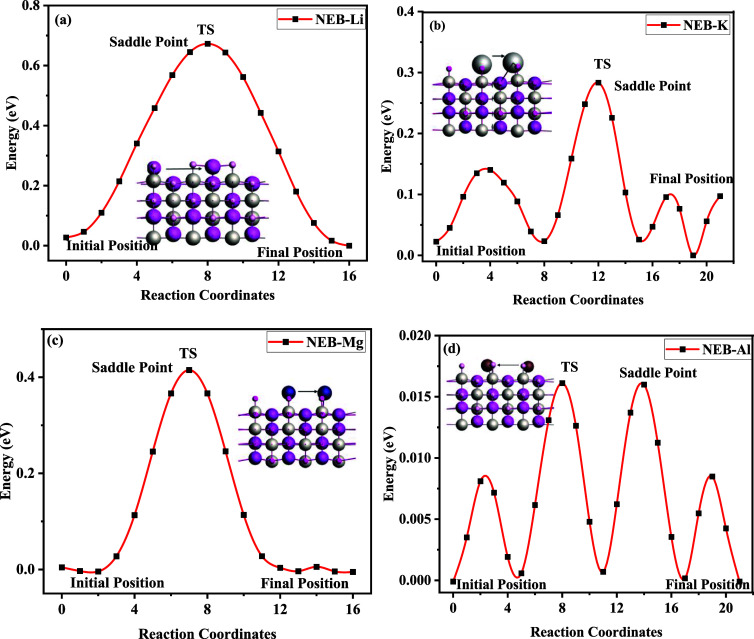
Diffusion Energy pathways of LiTiO2 for (a) **Li,** (b) K, (c) Mg, and­(d) Al.

The lithium-ion adsorption rate needs to be calculated, as the Li migration energy barrier depends on its concentration.^77–80^ We examined two limiting cases here, namely, the migration of a dilute metal ion and the migration of a dilute metal vacancy. For the dilute Li ion case, one Li ion is adsorbed on a supercell of the host, while for the dilute Li vacancy case, one Li ion is removed from the supercell of the fully saturated structure to generate the vacancy.^81^ To understand the dilute vacancy path, we considered Li, Mg, K, Al, and Ca by using the NEB calculations. In order to model the dilute Li vacancy, one Li was removed from the supercell of a fully lithiated structure, followed by the simulation across two different migration paths, as shown in Figure 7a. For the first path, the migration energy barrier was calculated as 1.4 eV, whereas its value for the second path was calculated as 1.8 eV, which is very small when compared to the reported Li-ion migration in graphene, silicon, and g-SiC.^82^ The smaller energy barrier promises very fast Li-ion diffusion across the host, which predicts an efficient charging process. A similar procedure was adopted in the case of Mg diffusion by considering two migration paths. The calculated migration energy barriers for path 1 and path 2 were 0.2 and 0.32 eV, respectively, which indicate a low energy barrier for the migration, as shown in Figure 7b. In the same way, for Ca ions, two migration paths were considered. To model the dilute Ca vacancy, one Ca was removed from the supercell for both paths, which yielded migration barrier values of 1.7 and 2.1 eV, respectively, as illustrated in Figure 7c. The NEB calculations were carried out for the K-ion dilute vacancy by removing the K ion from the supercell. The calculated values of the migration energy for the two paths were 1 and 1.1 eV, as shown in Figure 7d. Finally, the migration barrier for Al diffusion was calculated, providing values of 1.81 and 1.1 eV, as shown in Figure S4. The findings appeared consistent with the reported literature, as the energy barrier obtained for the fully loaded vacancy–dilute structure is comparatively higher than that of dilute structures.^55,83^

**Figure 7:**
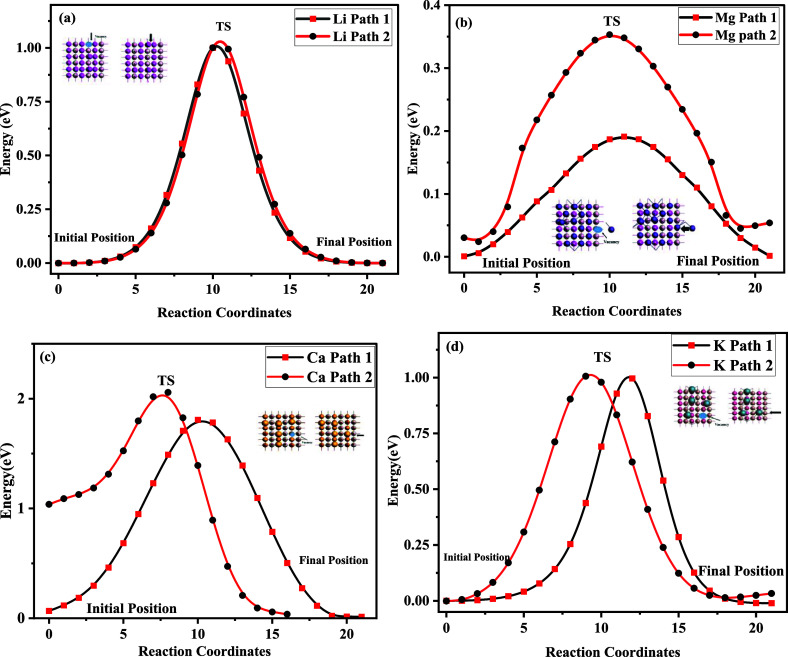
Diffusion energy and vacancy migration pathways of LiTiO_2_ for (a) Li, (b) Mg, (c) Ca, and (d) K. The dilute metal host structure and the dilute metal atom vacancy with a fully loaded host structure are utilized to compute these barriers.

### 3.10 Diffusion Coefficient and Ionic Conductivity

The diffusion coefficient for intercalating atoms in the host plays an important role in evaluating its anodic performance. The MD simulation was carried out in the temperature range of 300 K–900 K to calculate the diffusion coefficient. The temperature-dependent behavior of diffusivity and trajectory analysis uncovered that diffusion occurs more rapidly at elevated temperatures.^84^ The results provided in this work evidently show that LiTiO_2_ is a promising anode material for multivalent-ion batteries. The ionic conductivity (σ), related to the transport of metal atoms through the structure, is crucial for modeling the overall performance of the battery. The Arrhenius plot, drawn to examine the diffusion properties and related phenomena, is given in Figure 8. The calculated values of the diffusion coefficient are 7.84 × 10^–09^ m^2^/s, 0.83 × 10^–05^ m^2^/s, 0.66 × 10^– 9^ m^2^/s, 0.01 × 10^–11^ m^2^/s, and 7.05 × 10^–09^ m^2^/s for LIBs, MIBs, AIBs, CIBs, KIBs, respectively. The results show the lowest value of the diffusion coefficient in the case of CIBs, whereas the findings demonstrate the suitability of LiTiO_2_ as an anode material in multivalent batteries. The diffusion rates of Li, Mg, and Al atoms in the host materials are comparable to those reported for anode materials used in LIBs, MIBs, and AIBs.^85–88^ In the ongoing pursuit of identifying potential anode materials for LIBs^89–91^ and MIBs,^60,92,93^ researchers have developed numerous novel options. Nevertheless, further research endeavors are necessary to develop new anode materials offering the requisite electrochemical characteristics. In host LiTiO_2_, these figures show that Mg^2+^ ions have the highest diffusivity, followed by Li^+^ and K^+^. Due to its strong electrostatic interactions and higher ionic radius within the lattice framework, Ca^2+^ has a lower diffusion coefficient, which indicates sluggish ion transport. The final trend observed in the diffusion coefficient of several metallic ion species in LTO is governed primarily by the ionic radius, charge, and interaction strength of the migrating ion and the host lattice. The diffusion coefficient (*D*) varied in order of increasing values from 0.83 × 10^–5^ m^2^/s for Mg^2+^ (MIBs) to 0.07 × 10^–11^ m^2^/s for Ca^2+^ (CIBs). Again, Mg^2+^ has the highest *D* because it has the smallest size (72 pm) compared to Ca^2+^ (100 pm) and has moderate charge density, allowing for a relatively clean migration path. After analyzing the diffusion behavior of each ionic species, it was clear that Ca^2+^ suffered from slow diffusion because of its larger size and stronger interaction with electrons from the lattice oxygen atoms, inhibiting the mobility of the ion. Al^3+^ (AIBs) produced reasonable diffusion behavior (0.66 × 10^–9^ m^2^/s) likely because of both its charge and otherwise strong but localized binding, allowing Al^3+^ to channel through the migration space of certain host structures. K^+^ (KIBs) has a larger ionic radius (138 pm) but only a low charge, allowing it to produce moderate *D* values because of weaker electrostatic binding. Li^+^ (LIBs) produces a low *D* value (1.04 × 10^–12^ m^2^/s) in this case study because it is small and monovalent, and there may be strong binding at specific sites in the crystal structure of the LTO. The observed trend illustrates that while ionic size is certainly contributory, ionic mobility is not controlled solely by ionic size. Rather, the size, ion valency, and breadth of available topologies define the viability for ions to exhibit ionic transport behavior in electrode materials.^94^

**Figure 8:**
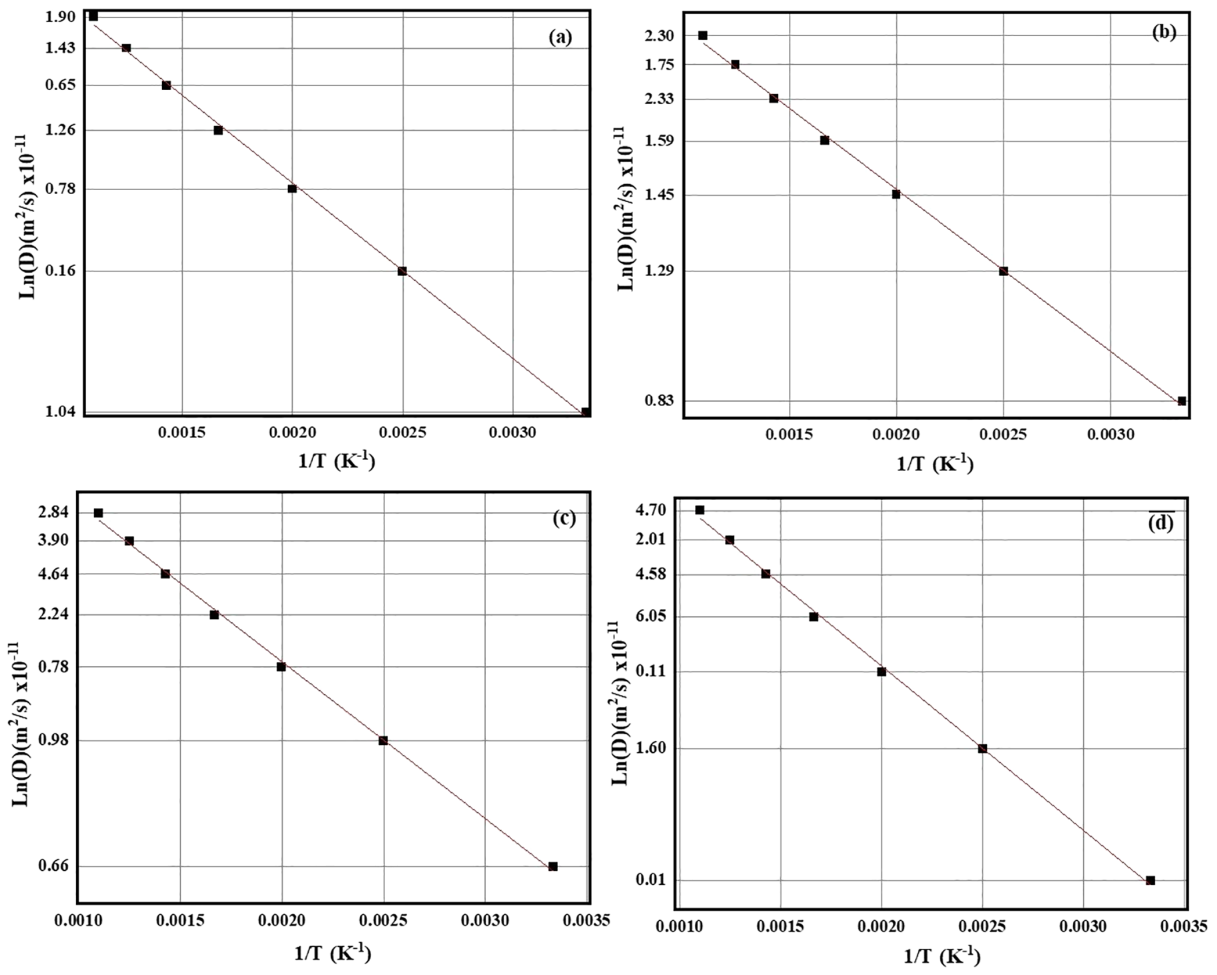
Diffusion coefficient of LTO after the intercalation of (a) Li, (b) Mg, (c) Ca, and (d) K and Al atoms (Figure S5).

The ionic conductivity is a crucial parameter to assess the ability of a material as an anode, as it determines how fast the metal ion can travel in the host material. The ionic conductivity is calculated as 2.32 × 10^–3^ Sm^–1^, 1.19 × 10^–2^ Sm^–1–^, 8.32 × 10^–2^ Sm–^1–^, 6.33 × 10^–3^ Sm^–1^, and 0.21 × 10^–2^ Sm^–1^ for LIBs, MIBs, CIBs, KIBs, and AIBs, respectively, as plotted in Figure 9.

**Figure 9:**
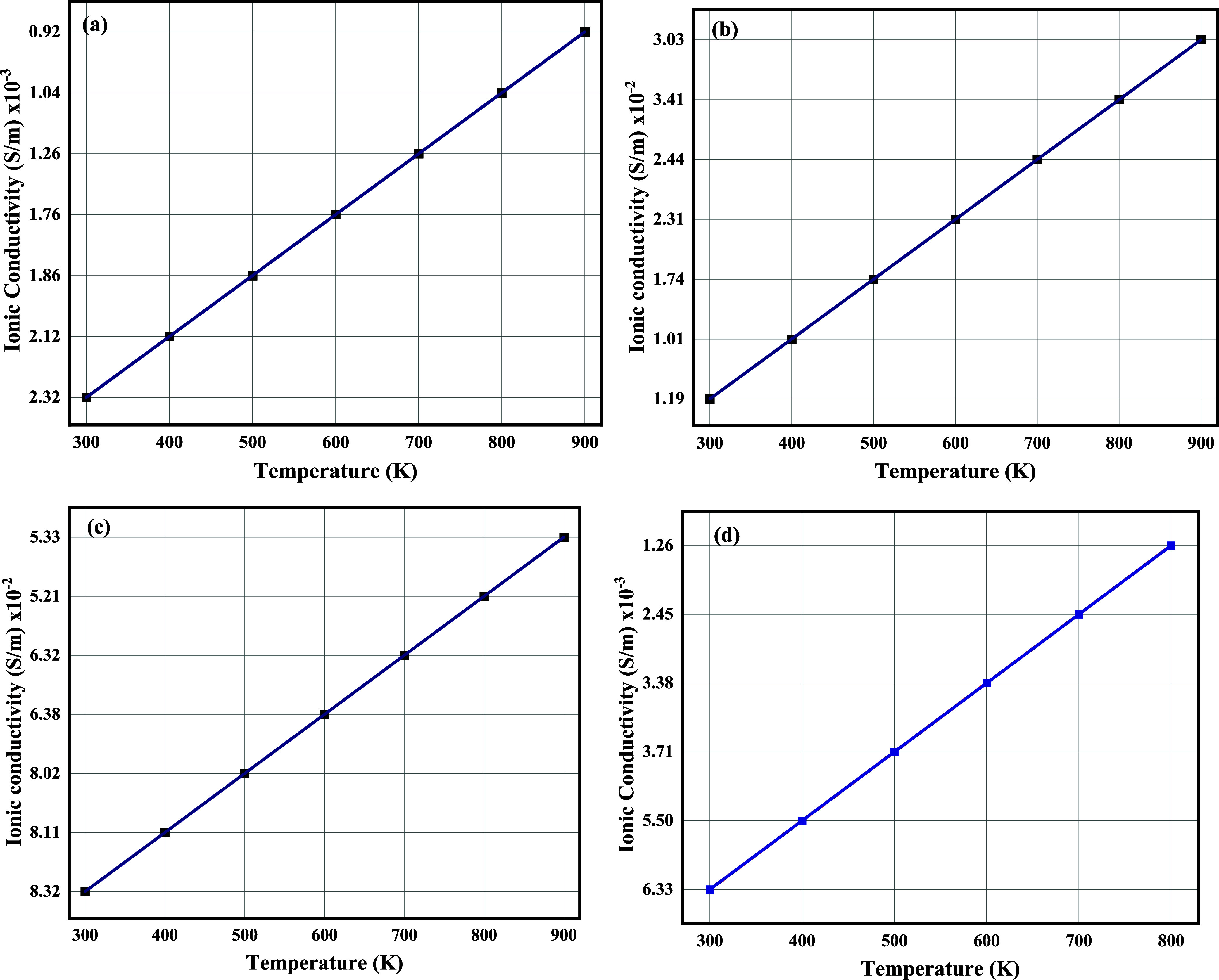
Ionic conductivity of (a) LTO after Li adsorption, (b) after Mg adsorption, (c) after Ca adsorption, (d) after K adsorption, and after Al adsorption (Figure S6).

LiTiO_2_ shows competitive ionic conductivity, which is similar to doped LTO systems and greater than that of anatase.^95^ Due to the high Coulombic interactions, Mg^2+^ ions typically show low conductivity. For example, the Chevrel phase Mo_6_S_8_ exhibits a conductivity of ∼10^–4^ S/m, and TiSe_2_ shows ∼10^–6^ S/m.^7^ Ca ions also typically show low conductivity; for instance, NaV_2_(PO_4_)_3_ shows ∼10^–6^ S/m, and MoO_3_ shows ∼10^–6^ S/m.^96^ In comparison, LiTiO_2_ shows comparatively high conductivity, which may suggest the possibility of enhancing Ca-ion transport in solid hosts. LiTiO_2_’s potential as a high-performance anode material for multivalent-ion batteries is shown by the better ionic conductivities of its multivalent ions, specifically for Mg^2+^ and Ca^2+^. Surprisingly, the conductivity of Ca^2+^ in LiTiO_2_ is higher compared to the majority of materials that have been identified, offering an appealing route for CIBs, which commonly show poor kinetics.

### 3.11 Hirshfeld Charge Analysis

The practical method for examining intermolecular interactions in solids is the Hirshfeld surface charge analysis.^97^ This process is distinct in its ability to compute and visualize the intercalant–host structure interactions in a crystal structure, covering distances from short to long, usually driven by donors and acceptors.^80^ These contacts are depicted through color-coded fingerprint plots and contour surfaces, with lengths shorter or longer than the sum of the van der Waals (vdW) radii shown by a spectrum ranging from red (shorter) to white and blue (longer).^80^ The transfer of charge between the intercalated atoms and the host structure before and after adsorption can be explored to shed light on battery operation and related electrochemical mechanisms. The calculated Hirshfeld charges on Li, Mg, Ca, K, and Al adsorption at LiTiO_2_ are illustrated in Figure S10, where blue indicates charge-deficient atoms and red denotes charge surplus. This points to visible charge collection or dissipation, which is important for analyzing the adsorption mechanism on the basis of charge dispersion between the adsorbed atoms (Li, Mg, K, Al, and Ca) and the host matrix. The positive charge on the metal atom after adsorption into LiTiO_2_ points to the transfer of electronic charge from the metal to the host, as illustrated in Figure S10.

### 3.12 Voltage–Current (*V*–*I*) Properties of LiTiO_2_

NEGF electron transport simulations are conducted on pure and Li-, Mg-, K-, Al-, and Ca- intercalated structures to further investigate the critical role of electron interaction in the growth of solid electrolyte interphase (SEI) layers. A circuit with two gold electrodes serving as wires to provide electrical power to the host material is illustrated in Figure S11. The NEGF setup with metal-atom-intercalated LiTiO_2_ is shown in Figure 10, whereas the *IV* characteristics obtained via applied voltage in the range of −5 to +5 V are shown in Figure 11. Pure LiTiO_2_ demonstrates a minimum current of 0.0009 μA, which increases to approximately 750 μA in the case of Li-, Mg-, Al-, Ca-, and K-atom-adsorbed structures.

**Figure 10:**
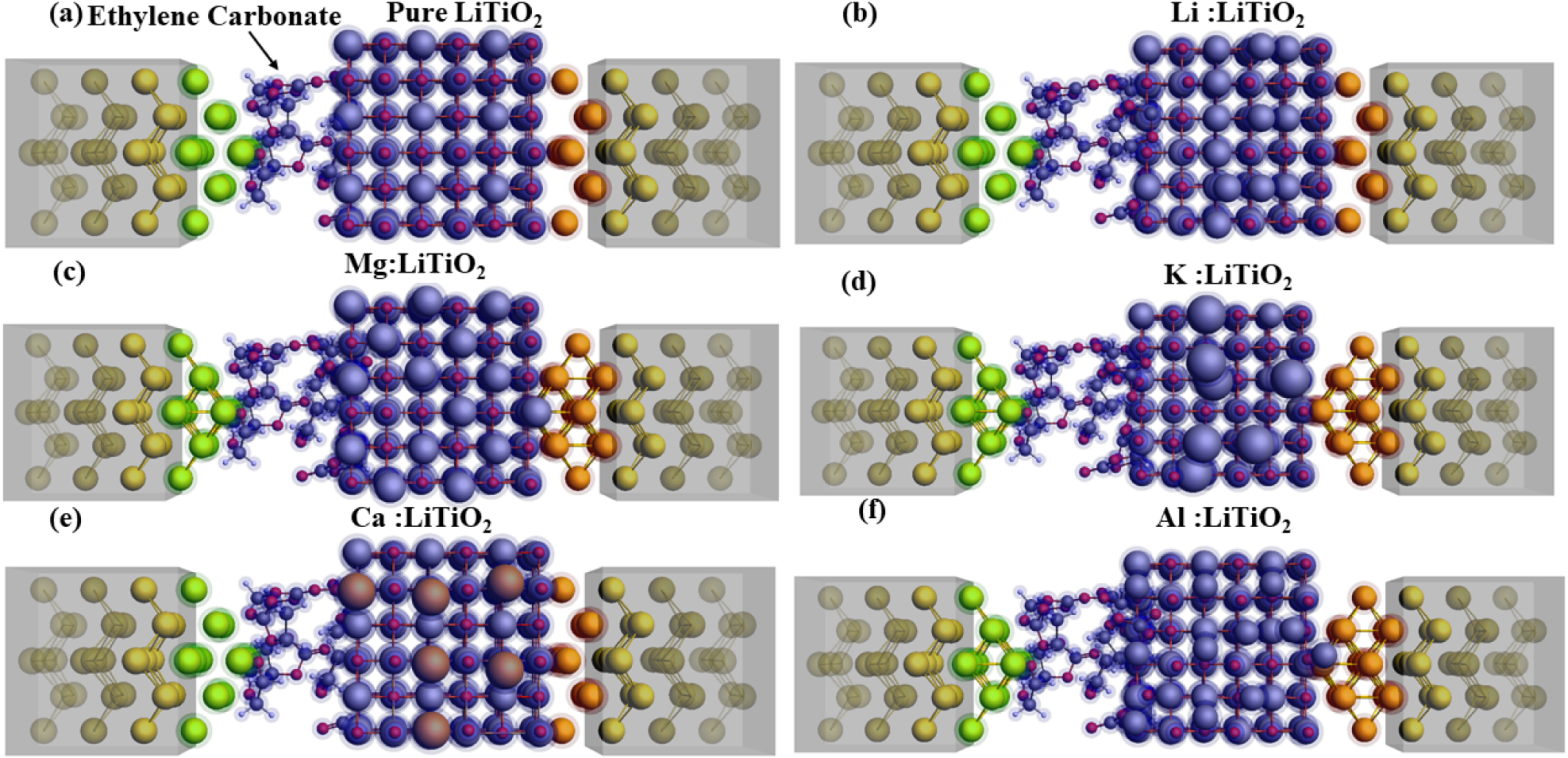
View of the NEGF setup where ethylene carbonate (EC) electrolyte is placed between the gold lead and the battery anode in the form of (a) pure LiTiO_2_, (b) Li-adsorbed LiTiO_2_, (c) Mg- adsorbed LiTiO_2_, (d) K-adsorbed LiTiO_2_, (e) Ca-adsorbed LiTiO_2_, and (f) Al-adsorbed LiTiO_2_. The proposed anode material is placed between gold leads.

**Figure 11:**
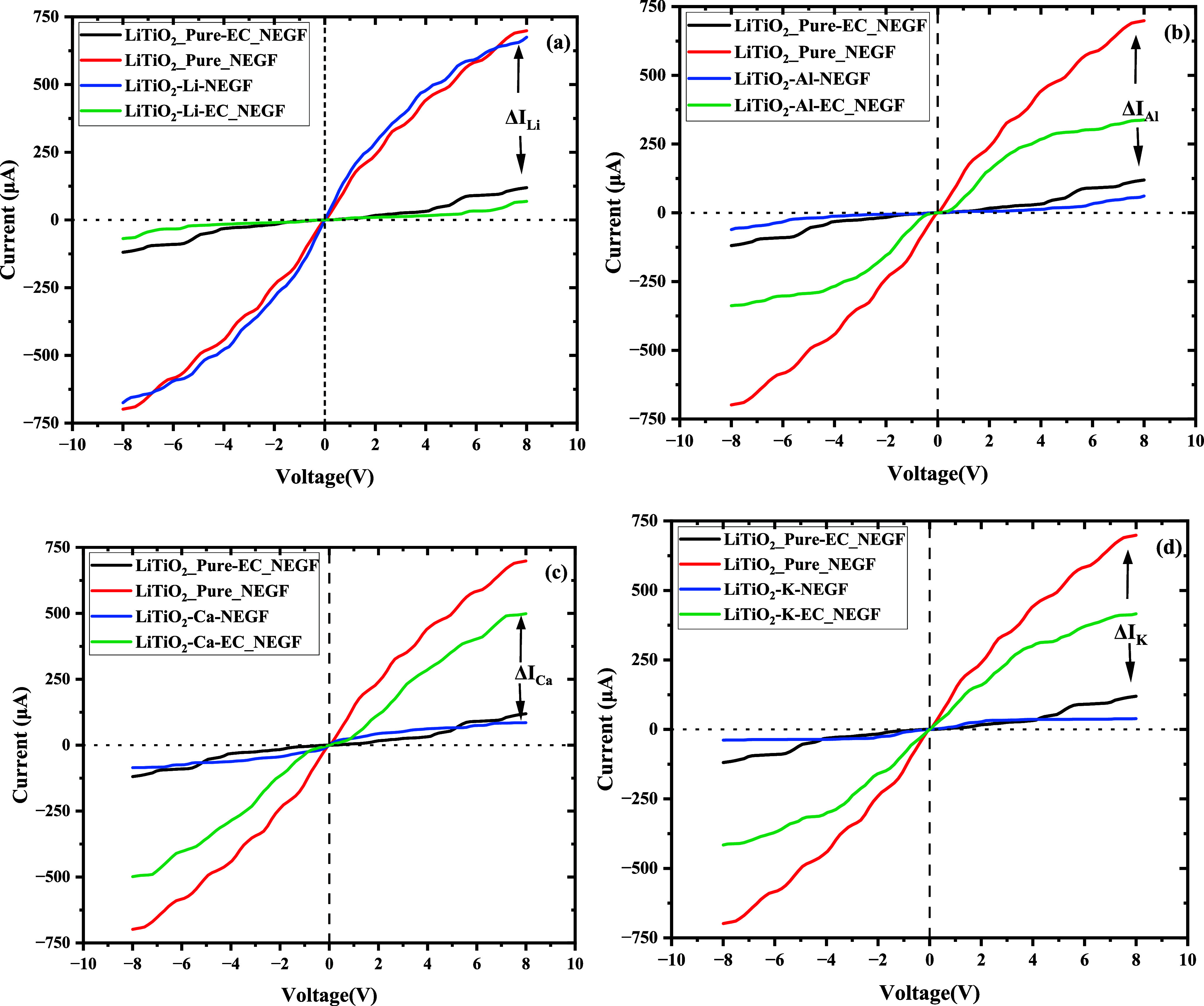
Current–Voltage (*IV*) characteristics showing: (a) comparison of *IV* curves of pure LiTiO_2_, Li-adsorbed LiTiO_2_, and EC + Li-adsorbed LiTiO_2_; (b) comparison of *IV* curves of pure LiTiO_2_, Al-adsorbed LiTiO_2_, and EC + Al-adsorbed LiTiO_2_; (c) comparison of *IV* curves of pure LiTiO_2_, Ca-adsorbed LiTiO_2_, and EC + Ca-adsorbed LiTiO_2_. (d) Comparison of *IV* curves of pure LiTiO_2_, K-adsorbed LiTiO_2_, and EC + K-adsorbed LiTiO_2_ and comparison of *IV* curves of pure LiTiO_2_, Mg-adsorbed LiTiO_2_, and EC + Mg-adsorbed LiTiO2 in Figure S7. The change in current with and without EC is shown by the Δ*I*.

The electron transport is studied for pure Li-, Mg-, Al-, Ca-, and K-adsorbed on LiTiO_2_ in the presence of ethylene carbonate (EC) as an electrolyte. The analysis of *IV* curves revealed a drop in current in the presence of EC, which was previously observed to increase when adsorption of the metal atoms into the host LiTiO_2_ was carried out. The examination of the *IV* curves of the intercalated host indicates that electron transfer processes are responsible for the current drop. The drop in current indicates electron donation, which points to the formation of a SEI layer on the surface of the anode material. The charge transfer and formation of the SEI layer assist the transport of metal ions across the structure of the rechargeable battery.^98^ Thus, the current passing through the electrode during the electrochemical reaction can be written as eq 10.

10 $$I_{\mathrm{ch}}=I_{\mathrm{SEI}}+I_{\mathrm{tran}}$$

While SEI is the currently related to SEI generation processes, the adsorption of the ions depends on the transfer charge current (*I*_tran_) in negative electrodes. The majority of the current is required for the primary electrochemical storage process to take place.^99^ Similarly, electrons from the EC electrolyte on the surface of the LiTiO_2_ assist in the formation of the SEI layer. In order to speed up the development of the stability of the SEI layer, as well as develop cost-effective and efficient metal-ion batteries, control over the carriers’ transport and currents may be helpful.^100^

## 4 Summary

This work employs first-principles investigations to calculate the potential of lithium titanate oxide (LiTiO_2_, LTO) as an anode material for multivalent-ion batteries, including lithium-ion (LIBs), aluminum-ion (AIBs), magnesium-ion (MIBs), calcium-ion (CIBs), and potassium-ion (KIBs) batteries. The research focuses on key anodic properties such as structural stability, storage capacity, adsorption energy, and ion migration dynamics. Adsorption of Li, Mg, Ca, K, and Al on LTO was analyzed, revealing exothermic reactions that confirm its suitability for battery applications. Storage capacities were calculated as 240 mAhg^–1^ (LIBs), 1131 mAhg^–1^ (AIBs), 1302 mAhg^–1^ (MIBs), 411 mAhg^–1^ (CIBs), and 171 mAhg^–1^ (KIBs), while open-circuit voltages (OCV) were determined as 3.31, 1.43, 1.09, 1.24, and 4.12 V, respectively, indicating compatibility, safety, and efficiency. Thermal stability was assessed using ab initio molecular dynamics (AIMD) simulations, with additional MD simulations at 300 and 900 K revealing diffusion coefficients of 1.04 × 10^–12^ m^2^/s (LIBs), 0.83 × 10^–5^ m^2^/s (MIBs), 0.66 × 10^–9^ m^2^/s (AIBs), 0.07 × 10^–11^ m^2^/s (CIBs), and 7.65 × 10^–9^ m^2^/s (KIBs). Ionic conductivity values were 2.32 × 10^–3^ Sm^–1^ (LIBs), 1.19 × 10^–2^ Sm^–1^ (MIBs), 8.32 × 10^–2^ Sm^–1^ (CIBs), 6.33 × 10^–3^ Sm^–1^ (KIBs), and 0.21 × 10^–2^ Sm^–1^ (AIBs), underscoring LTO’s rate performance. Cl-NEB simulations highlighted low energy barriers for ion migration of 0.52 eV (LIBs), 0.28 eV (KIBs), 0.43 eV (MIBs), 0.01 eV (AIBs), and 1.51 eV (CIBs), along with stable vacancy migration pathways. The solid electrolyte interphase (SEI) and electron transport properties were explored using the nonequilibrium Green’s function (NEGF) technique, analyzing current–voltage (*I*–*V*) characteristics. Collectively, the results demonstrate that LTO exhibits high storage capacity, thermal stability, and efficient ion transport, positioning it as a promising anode material for multivalent-ion batteries.

## Supplementary Material



## Data Availability

Not applicable
