# Supplementary material for: First-Principles Investigation of Lithium Titanate Oxide as an Anode Material in Li‑, Na‑, Mg‑, Ca-, and K‑Ion Batteries
Source: ACS Omega. 2025 Jul 24;10(30):33645–61. doi: 10.1021/acsomega.5c04533 (PMC12332574; doi:10.1021/acsomega.5c04533)
Supplement: Supplementary file 1 [file ao5c04533_si_001.pdf]

First-Principles Investigation of Lithium Titanate Oxide as an Electrode Material in Li, Na, Mg, Ca and K Ion Batteries

Abdul Majid[*,1], Ramla Ashfaq[1], Sawaira Tasawar[1], Mohammad Alkhedher[2], Sajjad Haider[3], Kamran Alam[4] , Hira Azhar Cheema[1]

[1]Department of Physics, University of Gujrat, Gujrat 50700, Pakistan

[2]Mechanical and Industrial Engineering Department, Abu Dhabi University, Abu Dhabi 59911, United Arab Emirates

[3]Chemical Engineering Department, College of Engineering, King Saud University, P.O. Box 800, Riyadh 11421, Saudi Arabia

[4]Department of Chemical Engineering, Materials Environment Sapienza, University of Rome, Roma, Italy

*Correspondence: abdulmajid40@uog.edu.pk ; kamran.alam@uniroma1.it

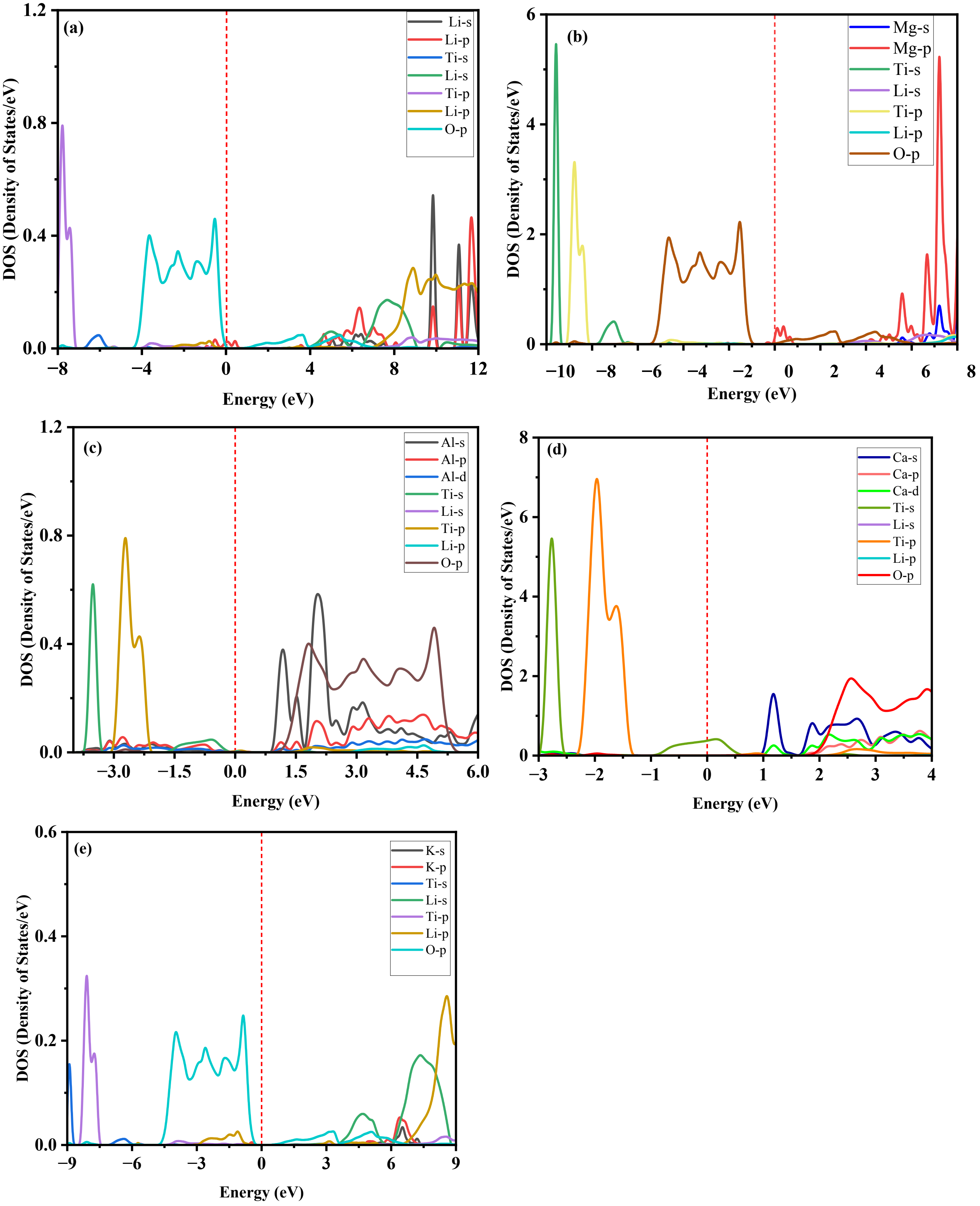
(a)
Li-s
Li-p
Ti-s
Li-s
Ti-p
Li-p
O-p
DOS (Density of States/eV)
Energy (eV)
(b)
Mg-s
Mg-p
Ti-s
Li-s
Ti-p
Li-p
O-p
DOS (Density of States/eV)
Energy (eV)
(c)
Al-s
Al-p
Al-d
Ti-s
Li-s
Ti-p
Li-p
O-p
DOS (Density of States/eV)
Energy (eV)
(d)
Ca-s
Ca-p
Ca-d
Ti-s
Li-s
Ti-p
Li-p
O-p
DOS (Density of States/eV)
Energy (eV)
(e)
K-s
K-p
Ti-s
Li-s
Ti-p
Li-p
O-p
DOS (Density of States/eV)
Energy (eV)

Figure-S1: The calculated DOS of $LiTiO_2$ after (a) lithium adsorption, (b) magnesium adsorption, (c) Aluminum adsorption, (d) Calcium absorption, and (e) Potassium adsorption. The Fermi level is adjusted to 0 eV.The Fermi level is adjusted to 0 eV.

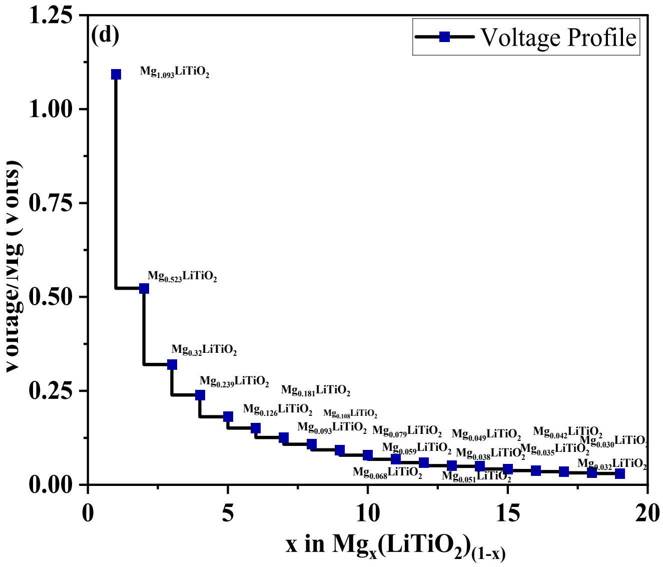

Figure-S2: The calculated voltage profiling of the $LiTiO_2$ plotted voltage/ metal-atoms in volts for Aluminum Ion batteries.

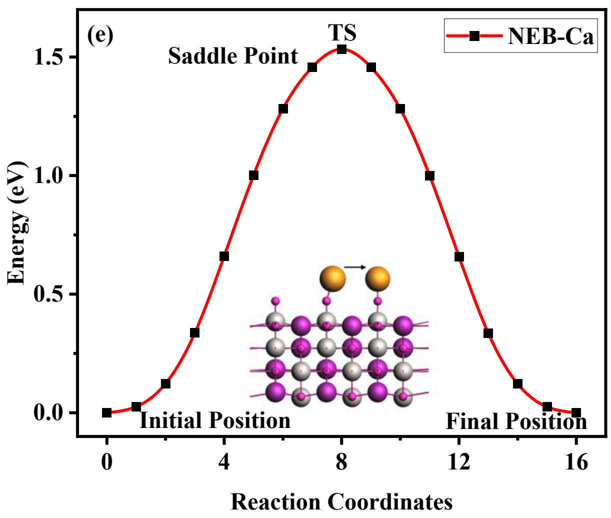

Figure S3. Diffusion Energy pathways of $Li_4Ti_5O_{12}$ for Calcium Ion Batteries

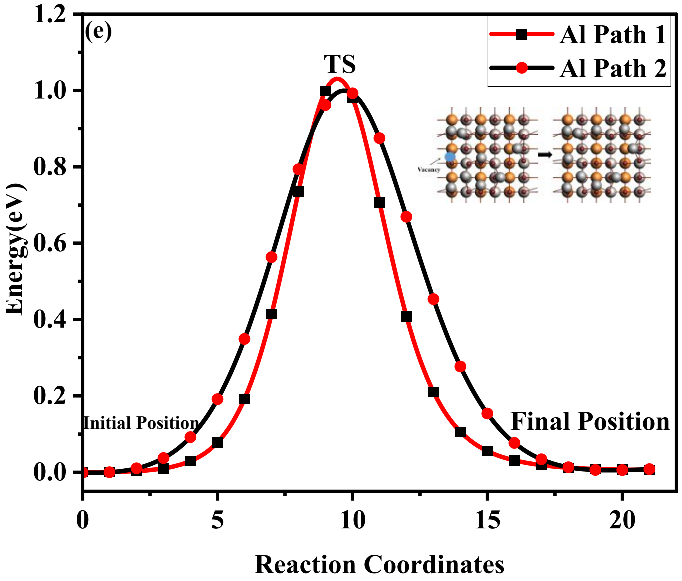

Figure-S4: Diffusion Energy Vacancy migration pathways of $LiTiO_2$ for Al. The dilute metal host structure and the dilute metal atom vacancy fully loaded host structure are utilized to compute these barriers.

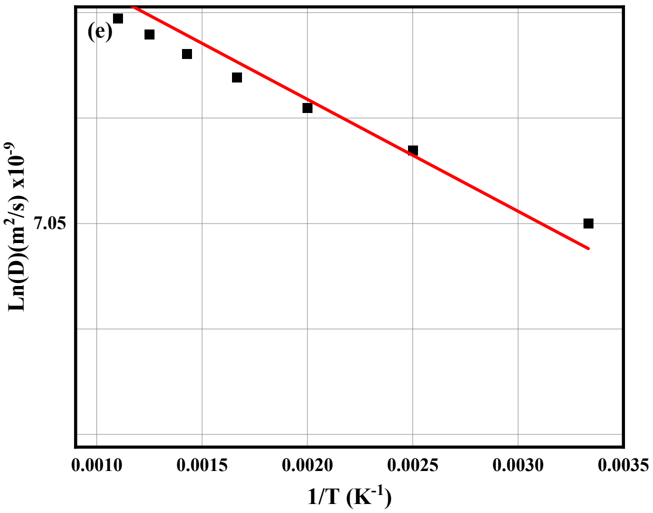

Figure-S5: Diffusion co-efficient of LTO after the intercalation of Al atoms.

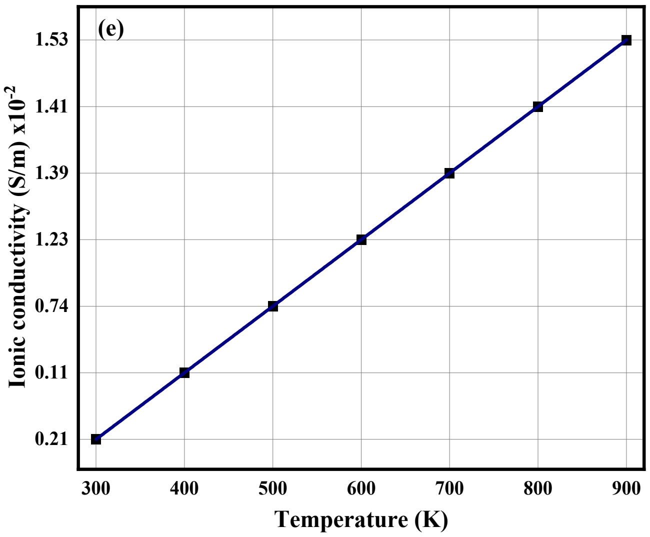

Figure-S6: The Ionic conductivity of Al adsorption.

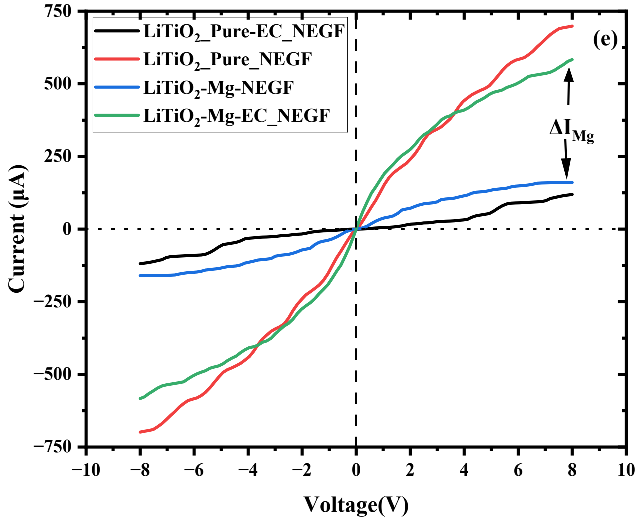

Figure-S7:The Current-Voltage (IV) characteristics showing comparison of IV curves of pure $LiTiO_2$, Mg adsorbed $LiTiO_2$ and EC +Mg adsorbed LiTiO2.The change in current with and without EC is shown.

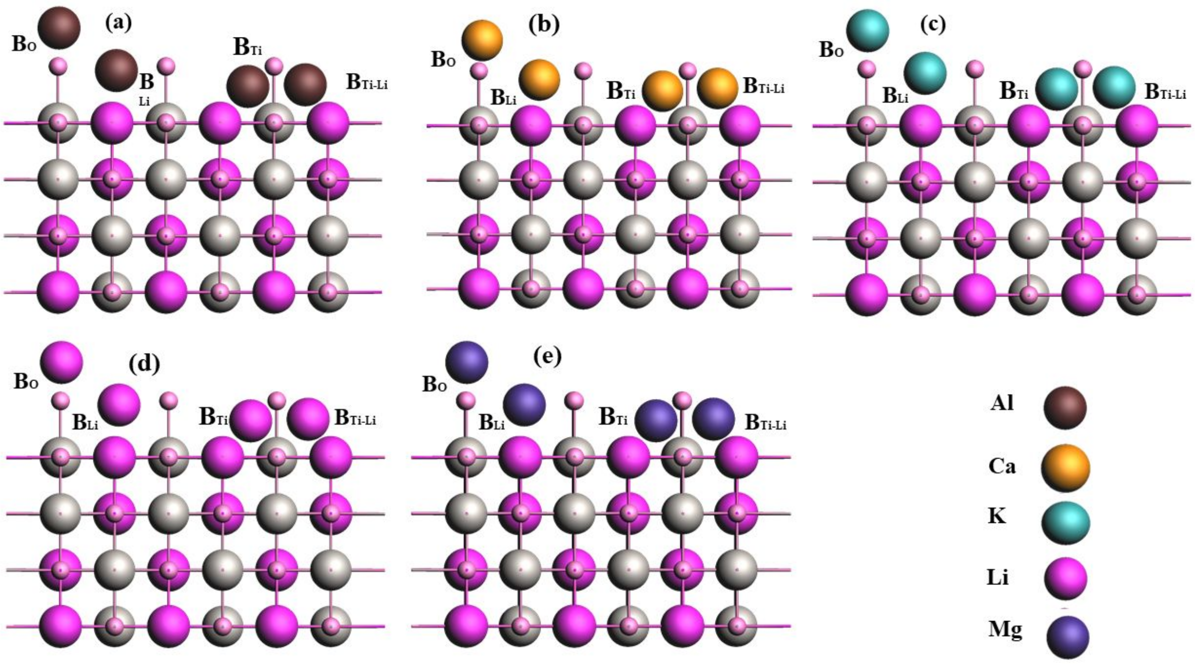

Figure-S8: The high symmetry sites for (a) Al, (b) Ca, (c) K, (d) Li and Mg adsorption in the host. The site B_O is interstitial site located near the O atoms of the host layers, site B_{Li} is situated close to the Li atom of either layer (upper) site B_{Ti} near the Ti atoms of the top layer. The site $B_{Ti\text{-}Li}$ is located between the Li atom and Ti atom of the layer.

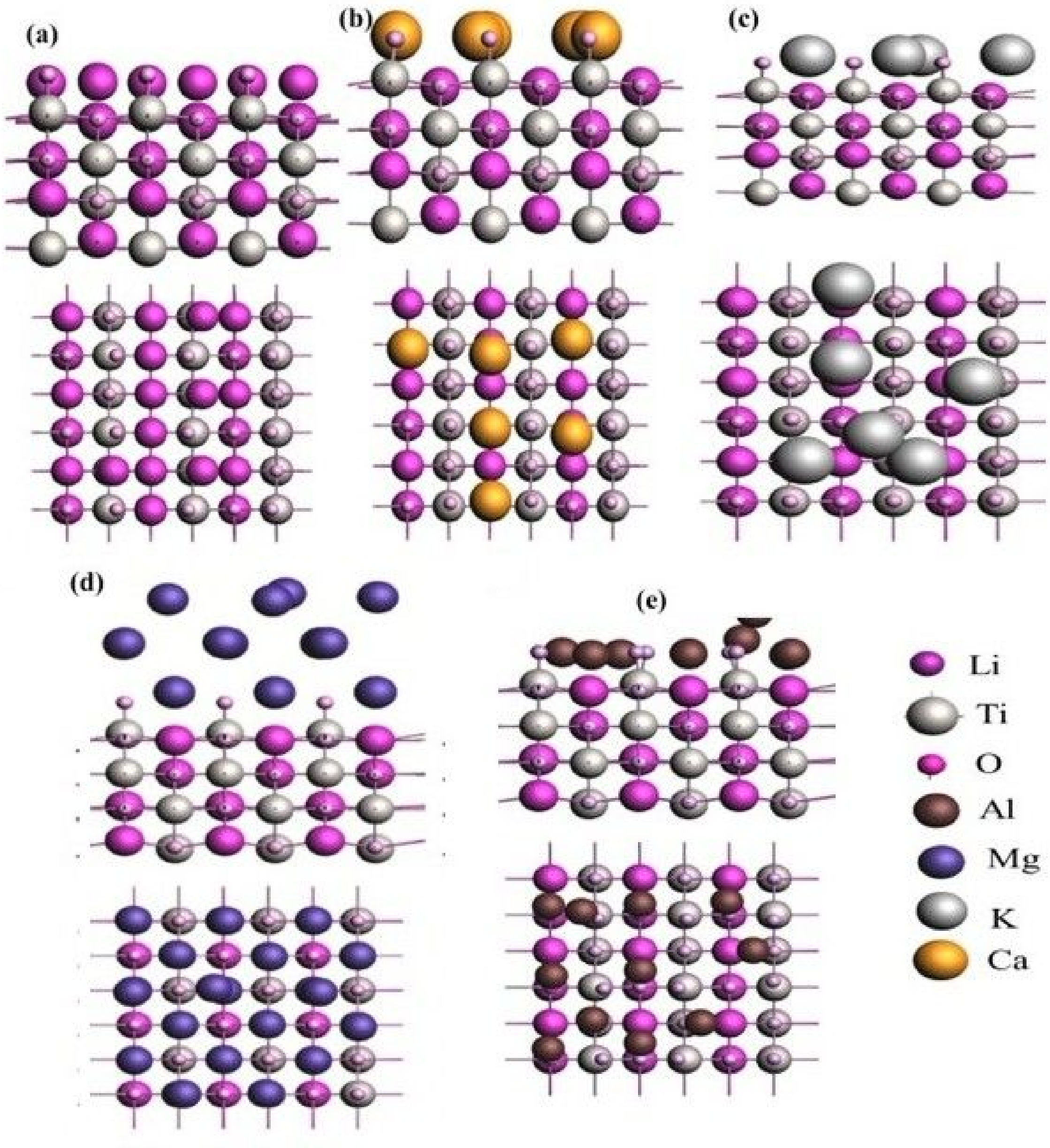

Figure S9: The $LiTiO_2$ with the maximum number of adsorbed (a) Li, (b) Ca, (c) K, (d) Mg and (e) Al atoms respectively.

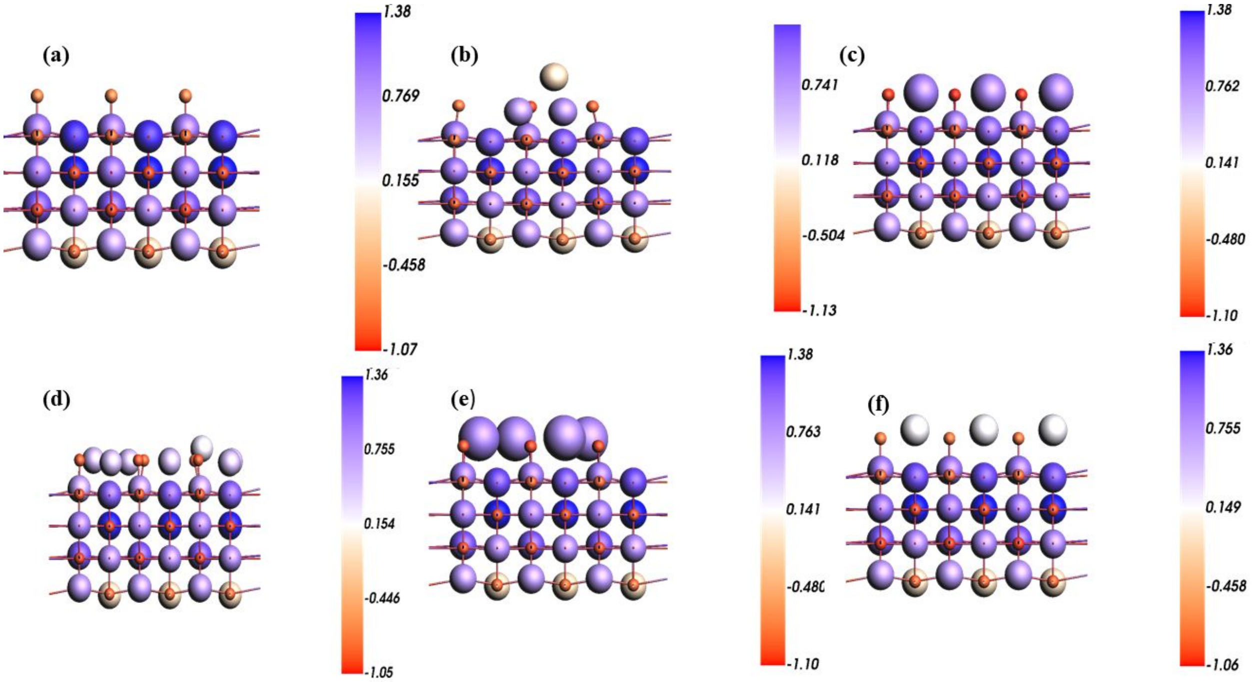

Figure S10: Hirshfeld charge analysis for (a) Pure $LiTiO_2$, (b) Li adsorbed $LiTiO_2$, (c) Mg adsorbed $LiTiO_2$, (d) Ca adsorbed $LiTiO_2$, (e) K adsorbed $LiTiO_2$ and (f) Al adsorbed $LiTiO_2$.

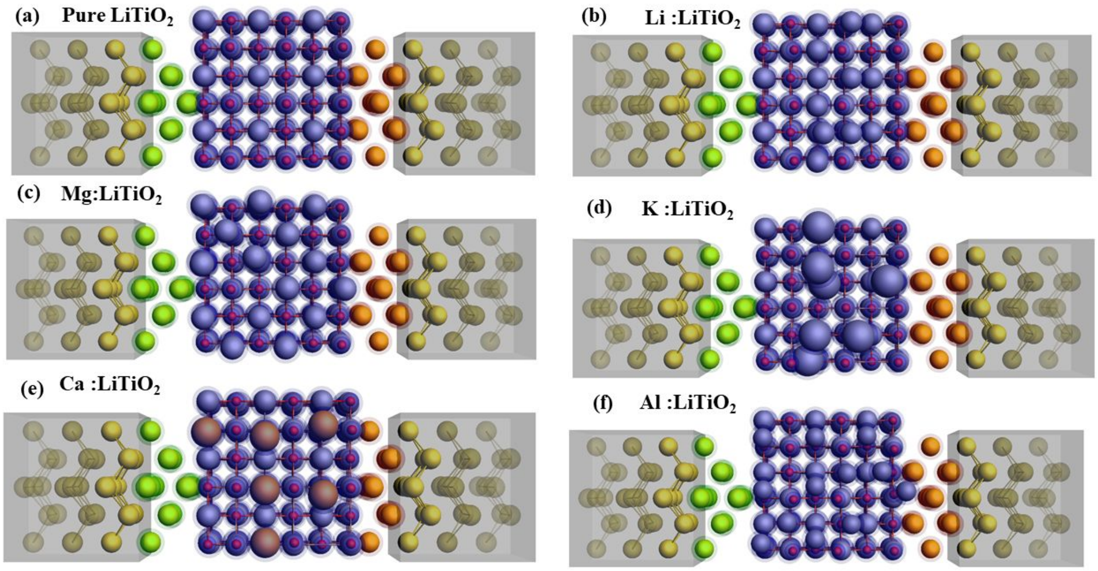

Figure S11: The view of NEGF setup of (a) pure $LiTiO_2$ (b) Li adsorbed $LiTiO_2$ (c) Mg adsorbed $LiTiO_2$ (d) K adsorbed $LiTiO_2$ (e) Ca adsorbed $LiTiO_2$ and (f) Al adsorbed $LiTiO_2$. The proposed anode material is placed between gold lead.

Table-S1: Adsorption energies for multivalent batteries on $LiTiO_2$.

No. of atoms	Adsorption energy (eV) for Li	Adsorption energy (eV) For K	Adsorption energy (eV) For Al	Adsorption energy (eV) For Ca	Adsorption energy (eV) For Mg	
**1**	-3.319	-2.483	-12.7868	-4.124	-2.187	**12:** -1.425
**2**	-3.183	-2.085	-9.94405	-2.785	-2.093	**13:** -1.372
**3**	-2.941	-1.601	-7.60347	-2.088	-1.923	**14:** -1.326
**4**	-2.523	-1.087	-6.89155	-1.474	-1.912	**15:** -1.286
**5**	-2.124	-0.548	-6.535	-0.954	-1.845	**16:** -1.247
**6**	-1.754	-0.286	-6.05013	-0.780	-1.818	**17:** -1.215
**7**	-1.728	-0.230	-5.72223	9.033	-1.768	**18:** -1.187
**8**	1.994		-5.08967	10.871	-1.728	**19:** -1.154
**9**	1.8123		-4.85702	17.172	-1.684	
**10**			-4.6619		-1.583	
**11**			-11.39398		-1.498	
